# “Time Saved” Calculations to Improve Decision-Making in Progressive Disease Studies

**DOI:** 10.14283/jpad.2024.64

**Published:** 2024-04-02

**Authors:** S. P. Dickson, B. Haaland, C. H. Mallinckrodt, B. Dubois, P. O’Keefe, M. Morgan, O. Peters, A. Fernández Santana, J. Harrison, Achim Schneeberger, S. Hendrix

**Affiliations:** 1 Pentara Corporation, 2261 E 3300 S, Millcreek, UT 84109 USA; 2 Alzheimer Research Center IM2A, GH Pitié-Salpêtrière, 83 Bd de l’Hôpital, 75013 Paris, France; 3 Department of Psychiatry and Neurosciences, CBF, Charité, Bonhoefferweg 3, 10117 Berlin, Germany; 4 Advantage Therapeutics, 195 NW 40th St, Miami, FL 33127 USA; 5 Scottish Brain Sciences, Gyleview House, 3 Redheughs Rigg, South Gyle, Edinburgh, EH12 9DQ UK

**Keywords:** Alzheimer’s disease, time saved, AFFITOPE® AD02, IMM-AD04, disease modifying agents

## Abstract

**Background:**

Disease modifying therapies (DMTs) may be most beneficial in early disease, when progression is slow and changes small, with clinical relevance difficult to interpret.

**Objectives:**

Time component tests (TCTs) translate differences between treatments from mean change, vertical distance between longitudinal trajectories, into intuitively understood time saved, horizontal distance between trajectories, which can be readily combined across endpoints in a global TCT (gTCT).

**Design:**

The value of composites, time savings estimates, and combination scores to optimize measurement and interpretation of DMTs are demonstrated, along with construction details and simulation studies.

**Setting:**

TCT methods were applied to a randomized phase II clinical trial.

**Participants:**

Patients with early Alzheimer’s disease (N=332).

**Intervention:**

Three treatment groups with AFFITOPE® AD02 and two control groups with aluminum oxyhydroxide, AD04.

**Measurements:**

The co-primary efficacy outcomes were an adapted ADAS-Cog (aADAS) and adapted ADCS-ADL (aADL), which were optimized composite scales specific to cognitive and functional domains. A composite based on these two scores was the study’s prespecified primary outcome. The CDR-sb and standard non-adapted ADCS-ADL and ADAS-Cog scales were prespecified secondary outcomes.

**Results:**

The AD04 2 mg group showed some statistically significant effects compared with other study arms. It is unclear whether the observed 3.8-point difference on the composite is clinically meaningful. TCT results show a time savings of 11 months in an 18-month study with AD04 2 mg.

**Conclusion:**

The relevance of 11 months saved is more universally understood than a mean difference of 3.8 points in the composite outcome. These results suggest that a combination of a composite approach and a time savings interpretation offers a powerful approach for detecting and interpreting disease modifying effects.

**Electronic Supplementary Material:**

Supplementary material is available for this article at 10.14283/jpad.2024.64 and is accessible for authorized users.

## Background

**Figure 1:**
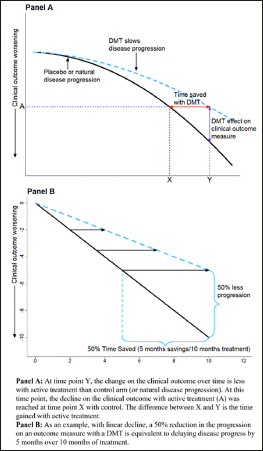
Examples of a disease-modifying effect with treatment delaying disease progression Panel A adapted from Dickson, et al. 2023, Journal of Prevention of Alzheimer’s Disease – https://creativecommons.org/licenses/by/4.0/

**D**isease modifying therapies (DMTs) are a focus of research for Alzheimer’s disease (AD). DMTs are expected to change the trajectory of disease progression and slow rates of clinical decline (1–3). DMTs may be most beneficial when treatment is begun early in disease to maintain higher levels of function longer and for patients to derive the most total benefit from treatment. However, progression is often slow in early disease and mean changes on placebo will be small. Even highly effective therapies will yield small effect sizes and the relevance of these differences are not easily understood (4, 5). The traditional treatment effect is the vertical difference between mean trajectories of treatment groups (see Figure 1, purple line). Understanding the clinical relevance of a difference between mean changes can be problematic when decline in the control arm is minimal.

Time-based metrics facilitate interpretation of specific endpoint differences by translating point-change differences into time saved with active treatment (6). Time saved is the horizontal difference between active and control for the change score observed at each visit in the active arm. It measures how long it takes for the placebo arm to progress as much as the active arm at a visit. Graphically, time saved is the horizontal difference between mean change trajectories rather than the traditional vertical difference between trajectories. Figure 1 provides an illustration of how a TCT converts absolute change (vertical distance) into time saved (horizontal distance) for simplified hypothetical disease trajectories. Panel A illustrates curves over the whole disease continuum, and Panel B illustrates a shorter duration in which progression is approximately linear. Converting differences to horizontal time savings can be particularly important with non-linear and “S” shaped trajectories, where ceiling and floor effects can make comparisons on the original scale challenging in early and late disease. The TCT constructions presented here are primarily based on the results of the original analysis, e.g., least squares means from a mixed model for repeated measures (MMRM). The TCTs function as a means to interpret the established primary analyses.

Only experienced researchers will understand the relevance of a particular change on the Alzheimer’s disease assessment scale–cognitive subscale (ADAS-Cog) or clinical dementia rating scale sum of boxes score (CDR-sb). However, the relevance of saving 6 months of progression will be readily understood as translating to preserving independence, longer participation in daily activities, and retaining relationships and sense of self.

Time savings allows comparison between outcomes within a study and between studies utilizing different endpoints. A key tenet of such cross-endpoint comparisons is an a priori expectation that endpoints are measuring a common underlying process, such as Alzheimer’s disease progression. Combining multiple estimates of time saved across clinical outcomes measuring different domains can improve power for detecting disease modifying treatment differences, but requires consistent effects across clinical outcomes, which is evidence of disease modification (DM), similar to a composite score (7–9).

In this investigation, we apply the TCT to a clinical trial in early AD with a composite endpoint as the prespecified primary outcome (10). One of the treatments evaluated in this trial was AD04, which is hypothesized to boost the immune system, ameliorate neuro-inflammation, and clear altered, oxidized lipids in the periphery, thereby restoring brain lipid homeostasis (10). Results suggested AD04 slowed hippocampal thinning and clinical decline. The additional analyses applied here are intended to: 1) illustrate the TCT methodology for individual items and the gTCT methodology for combining items in a proof-of-concept trial, 2) illustrate the ease in interpreting the clinical relevance of both individual TCT results and combined gTCT results, 3) demonstrate comparability of power for composite endpoints and gTCT approaches for measuring overall disease, and 4) use these new results to understand potential benefits of an experimental drug, AD04 2 mg for AD.

## Methods

### TCT Methodology

The TCT can be implemented on patient-level data or in a meta-analytic approach that utilizes trial-level summary statistics following the approach outlined in Dickson et al. (4). The meta-TCT takes least squares estimates from a primary analysis, such as MMRM, and uses a horizontal projection of mean changes on the active arm to connect to the time point in the control group at which the mean change in the control arm equaled the mean change on the active arm (4). A linear interpolation between visits is used to estimate the time point at which the mean change in the control group matches the mean change in the active group at the end of the study. This process is repeated for each assessment time. Estimated time saved is shown by the difference on the x-axis (time) between the horizontally connected points. Refer again to Figure 1 for an illustration. Note that the TCT construction used here does not make any assumptions on the linearity of the overall trajectory, beyond the linear interpolation between scheduled follow-ups. Details of TCT construction and standard error estimation are provided in Supplemental Material (Appendix, Technical Methods).

The time savings assessed from individual scales can be combined across multiple outcomes into a single inference using a method similar to a global statistical test (GST) that accounts for correlations between outcomes and is referred to as a global TCT (gTCT) (4, 11). Historically, composite scores have often been derived to measure the most progressive aspects of disease, resulting in a decline that is as linear as possible in a specific disease stage (12–14). Commonly, these composite scores are weighted linear combinations of individual items from one or more clinical scales, where the weightings are determined by the magnitude of progression on each item, with faster progressing items receiving greater weight (13). If these composite scores are nearly linear, they approximate a perfect time metric. For many composites, this has been accomplished by leaving out items that are not relevant at the targeted stage of disease, progressing too slowly, or have a low signal to noise ratio. For the gTCTs presented here, we adopt an approach minimizing the estimated variance of the weighted average – accounting for the variances of the individual endpoint time savings estimates as well as their inter-dependencies (Appendix, Technical Methods).

A TCT analysis can be applied to a composite scale in the same manner as an individual clinical scale. Our goal is to accurately measure disease progression and each of the three approaches (composite scores, TCTs, and gTCTs) addresses a particular limitation. Implementing all three together can provide an accurate and interpretable measurement of disease progression: first, restrict to progressive items in a composite for each domain, second convert each domain score to time in a TCT, and finally combine time savings estimates across domains in a gTCT calculation.

When time saved estimates are based on validated clinical scales, no additional outcome validation is required. Because time saved is derived from mean change analyses that are widely accepted, such as MMRM, the TCT does not require new or additional analyses or assumptions beyond the mapping of mean changes to time.

### Simulation Studies

A collection of simulation studies examining the type I error rate, power, bias, and standard error estimation of the proposed TCT and gTCT constructions is provided in the Technical Methods. Several themes are noteworthy in the first set of simulation studies summarized in Table A.1. First, examining the null hypothesis rejection rates for zero effect, we see no evidence of type I error inflation above the nominal 0.05, across individual endpoint TCTs and the gTCT. Second, while retaining type I error control when there is no treatment effect, the gTCT improves power when there are harmonious nonzero treatment effects on the endpoints whose evidence is being combined – particularly when the power for the individual endpoints is marginal (see the n=50 subjects per arm rows in Table A.1). The power benefits of the gTCT are strongest when the correlation between endpoints is weakest. Further, the results in Table A.1 indicate the proposed TCT and gTCT procedures for estimating time savings have low bias. The results in Table A.1 also suggest that power for comparisons of study arms on the time scale – via TCTs and gTCTs – may be slightly less than power on the original scale – via least squares means and GSTs. Assessments of type I error suggest that these time scale comparisons may be slightly conservative.

The second set of simulation studies whose results are shown in Table A.2 – assessing the quality of the proposed direct standard error estimation in comparison to a patient-wise bootstrap standard error estimation – suggest that the two approaches produce similar standard error estimates and inferences. However, these results suggest that the direct standard error estimates may be slightly conservative, in alignment with the results in the first set of simulation studies. If the computational resources are available, a patient-wise bootstrap standard error may be a high-quality alternative to the direct standard error estimates described above and in the Technical Methods section of the Supplementary Material.

The third set of simulation studies whose results are shown in Table A.3 examines scenarios where one would not a priori want to apply the gTCT construction that is presented in this manuscript. The gTCT approaches to combined time-savings presented here are constructed for measuring disease modifying treatment effects impacting multiple sequelae of disease. For treatments modifying disease downstream of its cause – the approaches presented here would not be expected to be optimal. Of course, in practice, hypotheses and preliminary data on disease modification can be mistaken, in which case the gTCT approach might be applied to pooling mixed null and non-null evidence – as this third set of simulation studies examines. As anticipated, the results in Table A.3 demonstrate that when the gTCT approach is applied to combining mixed evidence on null and non-null effects, power is reduced relative to directly testing the endpoint with the non-null effect and ignoring the endpoint with the null effect. The simulation results in Table A.3 also demonstrate that this power reduction due to pooling endpoints with conflicting evidence is stronger when the endpoints are more correlated and weaker when the endpoints are less correlated.

### Example clinical trial

The objective of phase II study AFF006 (NCT01117818) was to assess clinical activity of various doses and formulations of AFFITOPE® AD02 following its repeated subcutaneous administration to patients with early AD (10). The trial was a randomized, placebo-controlled, parallel group, double blind, multicenter trial performed at 32 sites in six countries. A total of 332 patients were enrolled and 283 patients completed the trial in three treatment groups with AD02 and two control groups with aluminum oxyhydroxide, here named IMM-AD04 (aka AD04). Each patient was randomly assigned to one of five groups: 1 mg IMM-AD04, 2 mg IMM-AD04, 25 µg AD02 (in two different formulations) and 75 *µ*g AD02 that also contained aluminum oxyhydroxide. The co-primary efficacy outcomes were the adapted ADAS-Cog (aADAS) and adapted Alzheimer’s disease cooperative study activities of daily living scale (ADCS-ADL – aADL), which are optimized composite scales specific to the cognitive and functional domains (13). A composite score (Composite) was the sum of these two scores, and this composite was the prespecified primary outcome of the study. The CDR-sb was a secondary outcome and was also included in the present analysis, along with the standard non-adapted ADCS-ADL and ADAS-Cog scales. The AFF006 trial was conducted in accordance with the Declaration of Helsinki, Good Clinical Practice, and local and international regulatory requirements. Subjects in the trial provided voluntary, written, informed consent. The trial was approved by an independent ethics committee, and obtained the committee’s approval before the trial was initiated.

Treatments were generally well tolerated and adverse events (AEs) were seen at similar rates across all treatment groups, with the exception that more injection site reactions were seen in the groups with a higher level of adjuvant (2 mg) than in controls. The control groups differed on aADAS and aADL and therefore, as defined in the statistical analysis plan, data from the two control groups were not pooled for the original, pre-planned analyses of AD02.

No statistically significant beneficial treatment effects were seen for the investigational compound, AD02. Unexpectedly, the 2 mg AD04 arm, a control arm, showed statistically significant effects (p<0.05) in comparison to at least one other study arm for several clinical outcomes, including: aADAS-Cog, aADL, Composite, ADAS-Cog, CDR-sb, and QOL-AD Caregiver as well as two biomarker outcomes: right and total hippocampal volume (10). In the 2 mg AD04 arm, 48% of patients had no decline in the Composite at 18 months compared to 17%–31% in the other groups. Disease progression in this trial’s other groups was overall consistent with historical placebo groups (13), albeit somewhat slower, possibly related to this trial’s comparator groups all receiving minimally active agents.

Using data from the AFF006 study, the aADAS, aADL, and CDR-sb were combined in a gTCT to estimate time savings for 2 mg AD04. Two additional gTCTs are also shown, combining CDR-sb and Composite as well as ADAS-Cog, ADCS-ADL, and CDR-sb. The first global test gTCT1 combines the benefit of the optimized composite for cognition and function and the global assessment, the second gTCT2 includes all 3 domains while retaining the primary endpoint as a component, and the third gTCT3 is included for its correspondence to historic clinical trials (Table 1). For each outcome, analyses were conducted via use of two control arm strategies and two patient populations. The two control arm strategies contrasted the 2 mg AD04 arm with: 1) the other study arms combined, and 2) the 1 mg AD04 arm. Each of these comparisons was conducted in two analysis sets: 1) all randomized patients, and 2) a subset of all randomized patients with mild AD defined as lower symptom severity at baseline (MMSE greater than or equal to 23).

**Table 1:** Changes from baseline and time saved for 2 mg AD04 compared with other arms combined

**Endpoint**	**Mean change (SE) to month 18**		**Mean difference (95% CI)**	**Time saved (months – 95% CI)**
	**2 mg AD04 arm**	**Other study arms combined**		
aADAS	3.0 (1.7)	6.7 (0.8)	−3.6 (−7.3, 0.0) p=0.0510	8.7 (−1.8, 19.3) p=0.1045
aADL	−8.3 (2.7)	−14.2 (1.4)	5.9 (−0.1, 11.9) p=0.0558	9.5 (1.2, 17.9) p=0.0251
CDR-sb	1.3 (0.4)	1.9 (0.2)	−0.6 (−1.4, 0.2) p=0.1491	6.6 (−1.8, 15.1) p=0.1248
Composite	2.4 (1.8)	6.3 (0.9)	−3.8 (−7.8, 0.1) p=0.0574	11.1 (2.2, 20.0) p=0.0143
ADAS-Cog	3.5 (1.4)	6.0 (0.7)	−2.5 (−5.5, 0.5) p=0.1036	6.2 (−4.4, 16.7) p=0.2503
ADCS-ADL	−5.9 (1.7)	−8.6 (0.8)	2.7 (−1.1, 6.4) p=0.1626	6.8 (−1.6, 15.3) p=0.1135
gTCT1: aADAS, aADL, and CDR-sb				8.3 (1.2, 15.5) p=0.0224
gTCT2: CDR-sb and Composite				8.7 (1.3, 16.1) p=0.0208
gTCT3: ADAS-Cog, ADCS-ADL, and CDR-sb				6.6 (−0.7, 14.0) p=0.0761

## Results

An overview of results from the all-patient cohort comparing the 2 mg AD04 vs the other study arms combined at month 18 is presented in Table 1. The difference between treatments in mean change from baseline to month 18 ranged from 0.6 points on the CDR-sb to 5.9 points on aADL. To interpret these results directly, one must have in-depth knowledge of the scales. Moreover, even with scale knowledge, it is hard to know if these treatment differences represent clinically meaningful benefit. In contrast, time savings were 8.7 months on the aADAS, 9.5 months on aADL, 6.6 month on CDR-sb, and 11.1 months on the Composite during this 18-month clinical trial. Using time saved, it is straightforward to understand which outcomes had the largest treatment benefit, and it is easier to understand if that benefit is meaningful.

**Figure 2:**
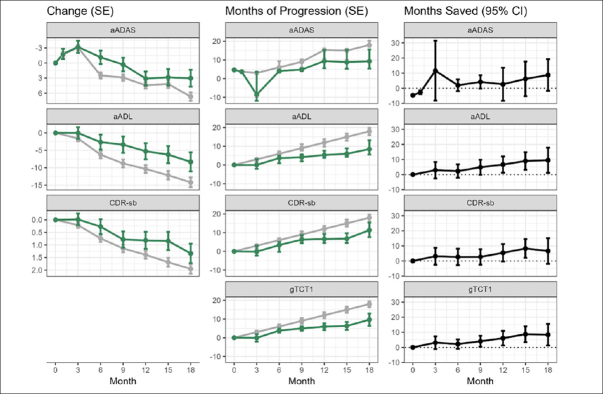
Trajectories over follow-up time by endpoint in the full patient cohort 2 mg AD04 arm in green, other study arms combined in gray; left panels show patient-level endpoints on original scale; middle panels show endpoints on TCT (or gTCT) scale; right panels show differences between study arms on TCT (or gTCT) scale (gTCT1: aADAS, aADL, and CDR-sb, gTCT2: CDR-sb and Composite, gTCT3: ADAS-Cog, ADCS-ADL, and CDR-sb).

Results for aADAS, aADL, CDR-sb, Composite, and gTCT1 are depicted in Figure 2. The left panels show mean endpoint trajectories over time on the original scale, the middle panels show TCTs (placebo progression time) over follow-up time, and the right panels show time saved with active treatment (i.e., the horizontal difference between treatment arms at each time point). Note that the gTCTs’ native scale is disease-time, so only the right panels are shown. Time saved was similar across endpoints in the mild subset with more time savings compared to the all-patient cohort; and results were similar across endpoints using either the 1 mg AD04 arm only as control or using all other arms combined as the control. Observed effects were stronger for the mild subset and slightly stronger when all other arms were combined as the control instead of just using 1 mg AD04 as the control. Results for the additional outcome variables and for combinations of all patients and mild Alzheimer’s subgroup (baseline MMSE ≥20) as well as 2 mg AD04 vs. other study arms combined and 2 mg AD04 vs. 1 mg AD04 at 18, 12, and 6 months are provided in the supplemental material (Appendix, Tables A.4–A.7 and Figures A.1–A.3). Interestingly, it is challenging to compare treatment effects in mild disease to treatment effects in both mild and moderate disease on either a point scale or a percent slowing scale, due to better statistical power to detect differences in later disease compared to earlier; however, comparing treatment effect estimates on a time scale is a fair standard for comparison since ceiling and floor effects of the scales are no longer relevant on the time scale. If more time savings is observed in early disease, this could be due to a slower accumulation of biological damage that is easier for an intervention to counter, which is consistent with disease modification.

**Figure 3:**
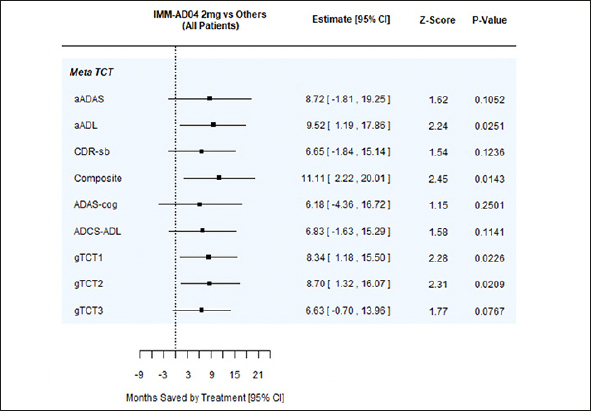
Forest plot summarizing time savings analyses at 18 months for full patient cohort, 2 mg AD04 vs. other study arms combined gTCT1: aADAS, aADL, and CDR-sb, gTCT2: CDR-sb and Composite, gTCT3: ADAS-Cog, ADCS-ADL, and CDR-sb

Time saved was also assessed with gTCTs that combined the time-saved results from individual scales. Time saved on the gTCTs was 6 months to nearly 9 months. When each of the component endpoints in a gTCT provide harmonious evidence of treatment differences, the gTCT can provide stronger evidence than individual endpoints. Consider for example, aADAS, aADL, and CDR-sb. As individual endpoints, their TCTs provide similar evidence of disease time saving, but only aADL achieves statistical significance at p<0.05. When these endpoints are combined into gTCT1, the overall evidence of time savings is even stronger. Another example is gTCT2, combining CDR-sb and Composite for 2mg AD04 vs. 1mg AD04 in patients with mild disease. Neither of the component endpoints (CDR-sb and Composite) achieve statistical significance, but gTCT2 has p=0.0394 (Table A.4 and Table A.7). A forest plot summarizing the time savings analyses at 18 months in the full patient cohort for 2mg AD04 vs. the other study arms combined is shown in Figure 3.

## Discussion

DMTs should be prescribed early in disease to delay progression as soon as possible, and maintain high levels of function, potentially over a longer duration of treatment. DMTs could have greater benefit at earlier stages of disease when biological damage may be easier to counter because it is accumulating more slowly.

Absolute differences between treatments in mean changes in endpoints during early disease are likely to be small even for highly effective treatments over typical durations of clinical trials. It can be difficult to contextualize small absolute differences between treatments when progression is also minimal in the control arm. Expressing results as percent slowing for active versus control can mitigate some of the difficulty in contextualizing results when progression is minimal in the control arm. Translating trial results into time saved can make results more interpretable to those who do not have extensive experience with the clinical trial scales.

Time saved is an easily understood metric because it is used in daily life. Moreover, time saved is of central importance in AD. Patients, families, and care partners want to know how long a treatment may be expected to maintain current lifestyle. Although converting treatment effects and progression to time saved has statistical challenges, the methods proposed above provide a foundation for improving interpretability and a means for combining evidence across endpoints. We note that it is not expected that TCT approaches will result in substantial power differences. The primary benefits of TCT transformations are transparent interpretation of treatment differences and straightforward pooling of evidence across endpoints, because they are all expressed on the same scale.

As illustrated in the example clinical trial, time saved provides a straightforward approach to comparing results across endpoints within a trial. This same flexibility facilitates comparison of results across trials with different outcomes. Multiple disease domains may be impacted simultaneously by DMTs, and these outcomes can be readily combined on the timescale in a gTCT by combining TCTs from individual scales. TCTs can also be applied to composite scores that have been derived to optimally measure disease progression. We hypothesize that constructing a composite first, before pooling time savings, may be optimal because irrelevant or low-signal endpoint items can be eliminated or down-weighted. We note that in Table 1 – as well as Supplemental Tables S1–S4 – the Composite, gTCT1, and gTCT2 demonstrate roughly equivalent strength of signal.

Liu-Seifert et al. (8) provides conditions under which a composite provides higher power than its component endpoints. Liu-Seifert et al. show that power for certain types of composites is at least as large as the minimum power of the components of the composite. Under certain conditions depending on the endpoints’ variability, correlation, and strength of effects, power for certain types of composites is at least as large as the maximum power of the components of the composite. To optimize power, a composite with optimized weights is typically best, e.g., ADCOMS (9). Again, Table 1 as well as Supplemental Tables S1–S4 demonstrate that optimized gTCTs nearly uniformly increase the strength of evidence relative to their component endpoints.

gTCTs are helpful because they combine evidence from endpoints on a common, clinically meaningful metric. gTCTs can provide better power than tests of their individual component endpoints, which is critical for these early small studies, when treatment effects are consistent across outcomes, as expected with DMTs. We note here that the GST methodology that the gTCT is based on is constructed for combining evidence on endpoints that are a priori expected to provide evidence on an entire process underlying the disease, e.g., Alzheimer’s disease progression. Clinical outcomes in progressive diseases often reflect distinct sequelae of disease progression and combining across outcomes representing more aspects of disease provides a stronger basis for disease modification. Composite endpoints, GSTs, and gTCTs are all approaches to combining evidence across endpoints. However, results based on composite endpoints and GSTs can be difficult to interpret. In contrast, gTCTs have a transparent time savings interpretation for combined evidence. We emphasize that the gTCTs constructed here preserve type I error control when there are not treatment effects on the endpoints being combined. If there are harmonious treatment effects across the endpoints across which evidence is being combined in the gTCT, then power can be increased.

We applied TCT methodology to the AFFITOPE® AD02 trial data to illustrate TCTs on individual scales, on composite scales, and when used globally in gTCTs that were combinations of the individual scales. Given the unexpected findings in this early phase trial, flexible analytic approaches were needed. Results were presented for multiple control arms (1 mg AD04 only as control or all arms other than 2 mg AD04 as control) and assessment of multiple patient subsets (all patients and mild patients only). Time saved during the 18-month trial varied from 3 to 12 months across endpoints, choice of control arm, and patient population, with most results clustering between 5 and 10 months saved. These results are on par with or better than results reported for monoclonal Aβ antibodies (5, 15, 16). Results were similar overall but favored 2 mg AD04 more strongly in the mild disease subgroup, consistent with a DMT, as well as with all other arms combined as the comparator, relative to 1 mg AD04 as the comparator, potentially due to a larger sample size. We emphasize that the trial results presented here were performed post-hoc with no adjustments for multiple comparisons, so the presented p-values should be considered nominal p-values and interpreted cautiously. The presented results require confirmation in future studies.

Results from these TCTs and gTCTs helped facilitate better understanding of the example trial’s results. The sponsor has used the results presented here to plan a phase 2 trial investigating AD04 in a placebo-controlled study in which the AD04 arm will be evaluated as the primary objective to evaluate the promising, post-hoc results from the AFF006 (NCT01117818) trial.

Given the relative newness of TCTs in clinical trials, several caveats are noteworthy. The present investigation utilized meta-TCTs, based on trial-level summary statistics. There are no presently published methods for constructing patient-level TCTs. However, construction of patient-level TCTs is an area of active research. Patient-level TCTs would allow usage of the full spectrum of statistical approaches and provide a direct theoretical basis for inference. Meta-TCTs have strengths including the transparency of their simple construction and their ability to use summary level data. Additionally, meta-TCTs based on progression models for repeated measures (PMRM) have been applied to clinical trial data (6). The PMRM methodology requires a few additional assumptions and can be more computationally burdensome in comparison to the TCTs presented here.

In addition, several methods of mapping mean changes to time can be envisioned. In certain situations, mapping choices may be important. For example, when mean changes over time are not monotonic, visit-wise assessments of time saved can show unusual results such as less disease time at a later timepoint than an earlier time point. Or, when a mean change on the active arm cannot be mapped to a time point on the control arm because the active arm mean change is outside the range of mean changes on control, time saved must be extrapolated beyond the time range of the trial. In segments of follow-up that are non-monotone or where there is very little information on the trajectories, then there could be substantial extrapolation. See, for example, the top row of panels in Figure 2. At 3-month follow-up, the active and placebo means are very similar (left panel). However, the active mean is slightly greater, so that there is no corresponding mean change from baseline on the placebo curve – except when extrapolated back in time. The confidence interval provides no evidence of time savings (right panel). Likewise, the approach presented here relies on linear interpolation between scheduled patient follow-ups. If follow-up times are sparse or the mean trajectory is strongly non-linear, then this linear interpolation may be a meaningful source of error too. We note that many of the complexities with TCT constructions and standard errors are driven by the non-parametric model adopted for the reference trajectory. If a smooth, parametric model were adopted for the reference trajectory, e.g., the PMRM model (6), then time translations and delta method standard errors would be simpler and more direct, albeit at the cost of flexibility and possible bias in trajectory estimates. Additionally, TCT constructions are adapted to progressive diseases and endpoints. If the reference trajectory is completely flat, or even improving, then mapping into TCTs will not be sensible. Although these are active areas of investigation that will lead to refinements, current approaches have worked well in diverse circumstances (4). While the time savings approach shares the facile interpretability of time-to-event analyses common in cancer studies, a key difference is that it does not rely on time to reach a single event or MCID change from baseline, but instead converts all changes from baseline simultaneously to a time on the reference trajectory. The presented TCT approaches are new and additional studies and comparisons to alternative and traditional approaches are needed before they can be established as possible primary endpoints in AD clinical trials.

In progressive diseases, time saved as measured via TCTs is intrinsically meaningful for expressing clinical trial outcomes in a manner that can be readily interpreted by diverse stakeholders, including patients, families, caregivers, and prescribers. TCTs do not entail additional scale validation. Use of TCTs also facilitates comparisons across outcomes within a trial and across trials with different outcomes. gTCTs are an important extension of the TCT methodology that reflect the inherent meaningfulness of DMTs and align the statistical analysis with the goal of disease modification. This approach allows DMTs to utilize a more powerful statistical test that performs well if multiple aspects of disease progression are similarly slowed with treatment. Presently available clinical endpoints are not sensitive enough to consistently measure individual level differences. However, global statistical testing approaches, such as the gTCT described here, could provide a path forward for constructing high resolution endpoints that can accurately identify slowing or lack of slowing of disease progression, analogous to using a high-powered telescope in astronomy. The TCT approach clarifies treatment benefits of AD04 in the example trial, by providing a transparent time savings interpretation and combining effects on time savings across multiple endpoints. This shift in emphasis from single outcomes and MCIDs to valuing disease slowing is critical to making progress in developing treatments for neurodegeneration.

### Electronic supplementary material

Appendix

## Notes

*Consent:* Subjects in the trial provided voluntary, written, informed consent.

## Notes

*Ethical standards:* The trial protocol, patient information, informed consent, and other relevant trial documents were submitted to independent ethics committees and approved.

## References

[1] Sabbagh MN, Richardson S, Relkin N (2008). Disease-modifying approaches to Alzheimer’s disease: Challenges and opportunities—Lessons from donepezil therapy. Alzheimer’s & Dementia.

[2] Cummings J (2017). Disease modification and Neuroprotection in neurodegenerative disorders. Translational Neurodegeneration.

[3] Gustavsson A, Pemberton-Ross P, Gomez Montero M, Hashim M, Thompson R (2020). Challenges in demonstrating the value of disease-modifying therapies for Alzheimer’s disease. Expert Review of Pharmacoeconomics & Outcomes Research.

[4] Dickson SP, Wessels AM, Dowsett SA (2023). ‘Time saved’ as a demonstration of clinical meaningfulness and illustrated using the Donanemab TRAILBLAZERALZ study findings. J Prev Alzheimers Dis.

[5] Van Dyck CH, Swanson CJ, Aisen P, Bateman RJ, Chen C, Gee M, Kanekiyo M, Li D, Reyderman L, Cohen S, Froelich L (2023). Lecanemab in early Alzheimer’s disease. New England Journal of Medicine.

[6] Raket LL (2022). Progression models for repeated measures: Estimating novel treatment effects in progressive diseases. Statistics in Medicine.

[7] Wessels A, Siemers E, Yu P (2015). A combined measure of cognition and function for clinical trials: the integrated Alzheimer’s disease rating scale (iADRS). J Prevent Alzheimers Dis.

[8] Liu-Seifert H, Andersen S, Case M (2017). Statistical properties of continuous composite scales and implications for drug development. J Biopharm Stat.

[9] Wang J, Logovinsky V, Hendrix SB (2016). ADCOMS: A composite clinical outcome for prodromal Alzheimer’s disease trials. J Neurol Neurosurg Psychiatry.

[10] Schneeberger A, Hendrix S, Mandler M (2015). Results from a phase II study to assess the clinical and immunological activity of AFFITOPE® AD02 in patients with early Alzheimer’s disease. J Prevent Alzheimers Dis.

[11] O’Brien, P.C.. Procedures for comparing samples with multiple endpoints. Biometrics, 1984;pp.1079–1087. 6534410

[12] Langbaum JB, Ellison NN, Caputo A (2020). The Alzheimer’s Prevention Initiative Composite Cognitive Test: A practical measure for tracking cognitive decline in preclinical Alzheimer’s disease. Alzheimers Res Ther.

[13] Hendrix S, Ellison N, Stanworth S (2015). Methodological Aspects of the Phase II Study AFF006 Evaluating Amyloid-beta-Targeting Vaccine AFFITOPE® AD02 in Early Alzheimer’s Disease - Prospective Use of Novel Composite Scales. J Prev Alzheimers Dis.

[14] Thomas RG, Albert M, Petersen RC, Aisen PS (2016). Longitudinal decline in mild-to-moderate Alzheimer’s disease: Analyses of placebo data from clinical trials. Alzheimer’s & Dementia.

[15] Budd Haeberlein S, Aisen PS, Barkhof F, Chalkias S, Chen T, Cohen S, Dent G, Hansson O, Harrison K, Von Hehn C, Iwatsubo T (2022). Two randomized phase 3 studies of aducanumab in early Alzheimer’s disease. J Prevent Alzheimers Dis.

[16] Sims JR, Zimmer JA, Evans CD, et al. Donanemab in early symptomatic Alzheimer disease: the TRAILBLAZER-ALZ 2 randomized clinical trial. JAMA. 2023 Jul 17. doi: 10.1001/jama.2023.13239. 10.1001/jama.2023.13239 PMC10352931 37459141

