# Supplementary material for: “Time Saved” Calculations to Improve Decision-Making in Progressive Disease Studies
Source: J Prev Alzheimers Dis. 2024 Apr 2;11(3):529–36. doi: 10.14283/jpad.2024.64 (PMC11060991; doi:10.14283/jpad.2024.64)
Supplement: Supplementary file 1 — Appendix [file 42414_2024_336_MOESM1_ESM.docx]

# Appendix

**Technical Methods**

*TCT estimation*

The TCTs presented here are based on trial-level summary statistics, such as least squares estimates from a primary analysis, e.g., an MMRM. Mean changes in the active arm are horizontally projected to the time point in the control group at which the mean change in the control arm equaled the mean change on the active arm (1). Mean changes in the control trajectory are linearly interpolated between visits to allow estimation of the time point at which each mean change in the active group matches the mean change in the control group. This process is repeated for each assessment time. For example, suppose we have a mean change score $x_{*}$ that we wish to map into a *monotone progressive* reference arm benchmark. If the nearest respective mean changes above and below $x_{*}$ on the reference trajectory are $\hat{x}_{1,\mathrm{ref}}$ and $\hat{x}_{2,\mathrm{ref}}$, with corresponding follow-up times $t_{1,\mathrm{ref}}$ and $t_{2,\mathrm{ref}}$, then the reference trajectory time corresponding to $x_{*}$ is $t_{*}=\frac{t_{2,\mathrm{ref}}-t_{1,\mathrm{ref}}}{\hat{x}_{2,\mathrm{ref}}-\hat{x}_{1,\mathrm{ref}}}\left( x_{*}-\hat{x}_{1,\mathrm{ref}} \right)+t_{1,\mathrm{ref}}$.

While the described TCT approach is intended for benchmarking with reference to a progressive trajectory, the reference trajectory is occasionally non-monotone, particularly in early follow-up or small sample size settings. In a case where the reference trajectory is non-monotone, some original scale values will map onto several possible times. Here, we adopt the convention of mapping to the maximum of the candidate times on the reference trajectory. This approach is conservative in the sense that it is inclined towards estimating slightly less time savings than the alternative candidate mappings. Additionally, there are certain situations where a time mapping off the range spanned by the reference trajectory is needed, for example, mapping standard errors near the beginning or end of follow-up (see below standard error mapping procedure). Here, we adopt the convention of time mapping off the range spanned by the reference trajectory via the line connecting the first visit – (0, 0) for change from baseline measures – and last visit measures. If the first and last visit reference trajectory values are not equal, then this convention allows all endpoint values to be mappable.

The standard errors from mean change analysis are converted onto the time scale by mapping the original scale estimates plus 1 standard error and the original scale estimates minus 1 standard error horizontally to the control reference trajectory in the same manner as the estimates themselves, to provide an upper and a lower TCT. The TCT standard error is then approximated as half the time difference between the upper and lower TCTs. The square of this standard error is equal to a weighted average of delta method variances (see Ver Hoef, 2012 for review of delta method [1]) over a time segment containing the mapped TCT estimate, with weights equaling the proportion of the interval from the lower TCT to the upper TCT in each time segment between patient follow-ups. An implication of this property is that if the original scale standard errors are small enough and the reference trajectory progressive enough, then many standard errors will either map into a single time segment – and the delta method standard error will be exact – or they will map into two adjacent time segments – whose slopes could both be regarded as sensible estimates of the reference slope in that time segment of follow-up.

For the delta method here, the transformation of interest $f(\cdot)$ is the “inverse” of the linearly interpolated reference trajectory and $f'(\cdot)$ is the “derivative” of this inverse. “Inverse” is in quotes for two reasons: (1) the reference trajectory may not be strictly monotone - in this case, our “inverse” is the largest of the possible solutions and (2) we may be requesting the “inverse” off of the range spanned by the reference trajectory - in this case, we use the inverse of the line connecting (0,0) to the reference mean at the last follow-up. “Derivative” is in quotes because we are taking the derivative of a piecewise linear function - this “derivative” is constant between the anchoring points, but does not exist at the anchoring points. Our approach to approximating the standard error on the time scale corresponds to first, approximating this derivative using an average over the time window from the “inverse” of one standard error above the estimate being converted to the “inverse” of one standard error below the estimate being converted, then applying the delta method directly.

The step-by-step procedure below generates the TCTs in step 4, their associated standard errors in step 5, and the time savings estimates along with standard errors in step 6 based on the least squares means by visit and study arm.

1. The TCT construction takes as inputs the least squares means by study arm and visit as well as their associated standard errors – one of the study arms is identified as the reference arm (ref) and the other as the active arm (act);
2. Construct a linear interpolation of the reference trajectory least squares means $\hat{x}_{1, \mathrm{ref}},\ldots,\hat{x}_{M, \mathrm{ref}}$ as a function of the scheduled follow-up times $t_{1},\ldots,t_{M}$ with the addition of the (0, 0) point corresponding to a mean change from baseline of zero at time zero;
3. Off the time range from time 0 to the last follow-up time $t_{M}$, extend the linear interpolation using the line through the two points (0, 0) and ${(t_{M}, \hat{x}}_{M, \mathrm{ref}})$ – reference arm mean at last follow-up – let  $\hat{g}(\cdot)$ denote the linearly interpolated and linearly extended reference trajectory;
4. For all means in both the reference and active arm, $\hat{x}_{1, \mathrm{ref}},\ldots,\hat{x}_{M, \mathrm{ref}}, \hat{x}_{1, \mathrm{act}},\ldots,\hat{x}_{M, \mathrm{act}}$, as well as the means plus 1 standard error and the means minus 1 standard error, $\hat{x}_{1, \mathrm{ref}}\pm{se}_{1, \mathrm{ref}},\ldots,\hat{x}_{M, \mathrm{ref}}\pm{se}_{M, \mathrm{ref}}, \hat{x}_{1, \mathrm{act}}\pm{se}_{1, \mathrm{act}},\ldots,\hat{x}_{M, \mathrm{act}}\pm{se}_{M, \mathrm{act}}$, find the maximum time at which $\hat{g}(\cdot)$ equals the target value: $tct=\max_{t} \{\hat{g}\left( t \right)=x\}$;
5. The mapped means in the reference and active arm, $\hat{tct}_{1, \mathrm{ref}},\ldots,\hat{tct}_{M, \mathrm{ref}}, \hat{tct}_{1, \mathrm{act}},\ldots,\hat{tct}_{M, \mathrm{act}}$, are the TCTs: $\hat{tct}=\max_{t} \{\hat{g}\left( t \right)=\hat{x}\}$;
6. The corresponding TCT standard errors are set as half the difference between the mapped upper and the mapped lower: $se_{tct}=0.5*|\max_{t} \{\hat{g}\left( t \right)=\hat{x}+se\}-\max_{t} \left\{ \hat{g}\left( t \right)=\hat{x}-se \right\}|$;
7. The TCT differences – time savings – are computed (for each follow-up time of interest) as $\hat{tct}_{\mathrm{diff}}=\hat{tct}_{\mathrm{ref}}-\hat{tct}_{\mathrm{act}}$, with corresponding standard deviation of the time savings $se_{tct \mathrm{diff}}=\sqrt{se_{tct, \mathrm{ref}}^{2}+se_{tct, \mathrm{act}}^{2}}$.

*gTCT estimation*

The gTCTs here minimize the estimated variance of the weighted average – accounting for the variances of the individual endpoint time savings estimates as well as their inter-dependencies. Here, the standard errors of the component TCTs are estimated according to the procedures above and their correlations are approximated as the correlations of residuals within each patient for the analysis models over endpoints (e.g., correlation of residuals from MMRM for endpoint 1 and residuals from MMRM for endpoint 2) – thereby defining the variance-covariance matrix of the component TCT estimates. In particular, for an estimated variance-covariance matrix of the time-savings across endpoints $\hat{\Sigma}$, we weight according to the solution to the convex program [2] $\min_{w} w'\hat{\Sigma}w$ subject to all the elements of the weight vector $w$ being non-negative and summing to 1. If the optimal weights are denoted $w_{*}$, then the standard error of the corresponding gTCT estimate is taken as $\sqrt{w_{*}'\hat{\Sigma}w_{*}}$.

The step-by-step procedure below generates the gTCT described above and its standard error in step 3 based on TCT time difference estimates and standard errors for the selected time point and the selected endpoints as well as analysis model residuals.

1. The gTCT construction takes as inputs (for each follow-up time of interest) the estimated time savings and associated standard error for each endpoint to be pooled $j=1, \ldots,J$: $\hat{tct}_{diff, j}$ and $se_{tct diff, j}$, as well as an estimated correlation matrix, $\hat{\Omega}:J\times J$, between the time savings estimates across endpoints.
   - Here, this correlation matrix is estimated based on the residuals from the individual MMRMs fit to change from baseline scores for each endpoint – adjusting for treatment arm, visit, and their interaction, as well as other model covariates.
2. The estimated variance-covariance matrix is based on the vector of standard errors and the correlation matrix, $\hat{\Sigma}=\mathrm{diag}\left\{ se_{tct diff, 1}, \ldots, se_{tct diff, J} \right\}*\hat{\Omega}*\mathrm{diag}\left\{ se_{tct diff, 1}, \ldots, se_{tct diff, J} \right\}$;
3. Let $w_{*}$ denote the solution to the quadratic program $\min_{w} w'\hat{\Sigma}w, subject to 1^{'}w=1 \mathrm{and} w\geq0$ (weights sum to 1 and weights are non-negative).
   - Here, this quadratic program is efficiently solved using the R package quadprog [3].
4. The gTCT estimate is $w_{*}^{'}*(\hat{tct}_{diff, 1}, \ldots,\hat{tct}_{diff, J})$ and its standard error is $\sqrt{w_{*}'\hat{\Sigma}w_{*}}.$

*Simulation studies assessing type I error, power, bias,* *and standard error estimation.*

A collection of simulation studies was performed to assess type I error, power, bias, and standard error estimation. In the simulation studies, we considered two hypothetical endpoints – roughly modeled after CDR-sb and ADAS-Cog – and combinations of linear/non-linear trajectories, effect sizes, and between endpoint correlation. For the linear trajectory scenarios, mean placebo change from baseline trajectories were given for time in months as:

$\mathrm{Endpoint} 1:y_{1}=-\frac{2.5}{18}*t and Endpoint 2:y_{2}=-\frac{7}{18}*t$.

For the non-linear trajectory scenarios, mean placebo change from baseline trajectories were given for time in months as:

$\mathrm{Endpoint} 1:y_{1}=-\frac{2.5}{2*18}*t-\frac{2.5}{2*{18}^{2}}*t^{2} and Endpoint 2:y_{2}=-\frac{7}{{18}^{2}}*t^{2}$.

For each simulation scenario, standard deviations for endpoints 1 and 2 are set as $SD_{1}=3 \mathrm{and} SD_{2}=8$, respectively. Intra-patient correlations, for a fixed endpoint across follow-up times, were set at 0.5 throughout simulation scenarios, with inter-endpoint correlations within patients set as 0, 0.3, or 0.6, across scenarios. Intra-patient correlations for distinct endpoints were taken as the product of the patient and endpoint correlations. For each simulation scenario, we consider follow-up 3-monthly through 18 months.

In the first set of simulation studies – assessing type I error, power, and bias, we examine three magnitudes of effect in the investigational arm 0, 0.25, and 0.50 – as a proportion of “time-shrinking”, $t_{\mathrm{investigational}}=(1-\mathrm{effect})*t$. Note that 0 corresponds to “no effect”, where the placebo and investigation arm mean trajectories are identical, for type I error estimation. Individual change from baseline measurements for each patient, across follow-up times and endpoints, were generated from a multivariate Gaussian distribution with mean and variance-covariance as described above, for 500 independent iterations. Analyses of simulated patient data mimicked the TCT and gTCT analyses presented here and described above for each simulation iteration. Table A.1 summarizes rejection rates for both TCT analyses and original scale analyses for the null hypothesis of no time savings at 18 months – type I error rate when effect is zero and power when effect is non-zero – as well as bias - $\left( average estimated time savings \right)-(true time savings)$ – across trajectory shapes, effects, correlations between endpoints, and sample sizes in each study arm. Note that the original scale global statistical tests (GSTs), combining evidence across $k$ endpoints on the original scale, are based on averaged Z-scores across endpoints with appropriately adjusted standard error accounting for the average correlation $\bar{\rho}$ between Z-scores across endpoints $\sqrt{(1+\left( k-1 \right)*\bar{\rho})/k}$.

In the second set of simulation studies – assessing the standard error estimation procedure described above in comparison to patient-wise bootstrap standard error estimation, we examine two effects in the investigational arm 0 and 0.25 – again as proportions of “time-shrinking”, $t_{\mathrm{investigational}}=(1-\mathrm{effect})*t$. Similar to the first set of simulation studies, individual change from baseline measurements for each patient, across follow-up times and endpoints, were generated from a multivariate Gaussian distribution with mean and variance-covariance as described above. Given the high computational burden of the patient-wise bootstrap standard errors, simulation results are based on 100 independent iterations, with 100 patient-wise bootstraps within each iteration. Inter-endpoint correlations within patients were set as 0.3 throughout the second set of simulation studies. Analyses of simulated patient data again mimicked the TCT and gTCT analyses presented in this manuscript. Table A.2 summarizes rejection rates and mean standard errors across simulation iterations for both the presented direct standard error estimates and patient-wise bootstrap standard error estimates.

In the third set of simulation studies – assessing type I error and power for mixed null and non-null treatment effects across endpoints, we examine a null effect (zero “time-shrinking”) for Endpoint 1 and two non-null effects in the investigational arm, 0.25 and 0.50. The remaining simulation settings in the third set parallel the first set of simulation settings. Table A.3 summarizes rejection rates for TCT and gTCT analyses at 18 months – type I error rate when effect is zero and power when effect is non-zero – across trajectory shapes, effects, correlations between endpoints, and sample sizes in each study arm.

| Trajectory shape | Effect – time shrinking | Correlation between endpoints | Sample size per arm | Rejection rate – TCT endpoint 1 | Rejection rate – TCT endpoint 2 | Rejection rate – gTCT | Rejection rate – original scale endpoint 1 | Rejection rate – original scale endpoint 2 | Rejection rate – original scale GST | Bias – endpoint 1 | Bias – endpoint 2 | Bias – gTCT |
| --- | --- | --- | --- | --- | --- | --- | --- | --- | --- | --- | --- | --- |
| Linear | 0 | 0 | 50 | 0.048 | 0.044 | 0.056 | 0.054 | 0.04 | 0.046 | -0.554 | -0.428 | -0.015 |
| Linear | 0 | 0 | 100 | 0.032 | 0.032 | 0.048 | 0.054 | 0.04 | 0.064 | -0.203 | -0.249 | -0.044 |
| Linear | 0 | 0 | 150 | 0.04 | 0.036 | 0.042 | 0.06 | 0.07 | 0.048 | -0.155 | -0.045 | 0.105 |
| Linear | 0 | 0 | 200 | 0.038 | 0.034 | 0.036 | 0.042 | 0.046 | 0.036 | -0.010 | 0.033 | 0.152 |
| Linear | 0 | 0 | 250 | 0.042 | 0.036 | 0.046 | 0.058 | 0.042 | 0.056 | -0.071 | -0.188 | 0.002 |
| Linear | 0 | 0.3 | 50 | 0.052 | 0.038 | 0.048 | 0.038 | 0.052 | 0.042 | -0.624 | -0.453 | -0.056 |
| Linear | 0 | 0.3 | 100 | 0.034 | 0.034 | 0.042 | 0.052 | 0.042 | 0.048 | -0.219 | -0.060 | 0.117 |
| Linear | 0 | 0.3 | 150 | 0.03 | 0.044 | 0.042 | 0.044 | 0.054 | 0.058 | -0.017 | -0.355 | -0.007 |
| Linear | 0 | 0.3 | 200 | 0.046 | 0.052 | 0.054 | 0.07 | 0.068 | 0.058 | 0.074 | -0.013 | 0.143 |
| Linear | 0 | 0.3 | 250 | 0.032 | 0.036 | 0.034 | 0.066 | 0.054 | 0.05 | 0.086 | -0.175 | 0.069 |
| Linear | 0 | 0.6 | 50 | 0.038 | 0.03 | 0.04 | 0.04 | 0.036 | 0.048 | -0.311 | -0.061 | 0.264 |
| Linear | 0 | 0.6 | 100 | 0.04 | 0.038 | 0.044 | 0.032 | 0.046 | 0.052 | -0.148 | -0.281 | 0.079 |
| Linear | 0 | 0.6 | 150 | 0.02 | 0.03 | 0.036 | 0.026 | 0.036 | 0.034 | -0.105 | -0.115 | 0.035 |
| Linear | 0 | 0.6 | 200 | 0.034 | 0.032 | 0.04 | 0.054 | 0.048 | 0.054 | -0.195 | -0.161 | 0.005 |
| Linear | 0 | 0.6 | 250 | 0.042 | 0.048 | 0.048 | 0.058 | 0.056 | 0.052 | -0.078 | -0.147 | 0.024 |
| Linear | 0.25 | 0 | 50 | 0.134 | 0.154 | 0.282 | 0.162 | 0.17 | 0.288 | -0.394 | -0.139 | -0.164 |
| Linear | 0.25 | 0 | 100 | 0.272 | 0.264 | 0.558 | 0.33 | 0.336 | 0.602 | 0.089 | 0.025 | 0.069 |
| Linear | 0.25 | 0 | 150 | 0.382 | 0.394 | 0.682 | 0.454 | 0.438 | 0.728 | -0.096 | -0.081 | -0.150 |
| Linear | 0.25 | 0 | 200 | 0.448 | 0.504 | 0.832 | 0.522 | 0.596 | 0.856 | -0.086 | -0.136 | -0.163 |
| Linear | 0.25 | 0 | 250 | 0.614 | 0.634 | 0.93 | 0.656 | 0.672 | 0.94 | 0.139 | 0.010 | 0.046 |
| Linear | 0.25 | 0.3 | 50 | 0.164 | 0.18 | 0.252 | 0.172 | 0.212 | 0.27 | -0.275 | -0.187 | -0.156 |
| Linear | 0.25 | 0.3 | 100 | 0.272 | 0.3 | 0.436 | 0.33 | 0.366 | 0.48 | 0.136 | 0.122 | 0.096 |
| Linear | 0.25 | 0.3 | 150 | 0.378 | 0.41 | 0.582 | 0.458 | 0.462 | 0.628 | -0.026 | -0.097 | -0.101 |
| Linear | 0.25 | 0.3 | 200 | 0.498 | 0.51 | 0.708 | 0.544 | 0.562 | 0.748 | -0.015 | -0.143 | -0.069 |
| Linear | 0.25 | 0.3 | 250 | 0.58 | 0.608 | 0.804 | 0.63 | 0.652 | 0.844 | 0.024 | -0.128 | -0.074 |
| Linear | 0.25 | 0.6 | 50 | 0.136 | 0.142 | 0.178 | 0.148 | 0.2 | 0.21 | -0.740 | -0.309 | -0.376 |
| Linear | 0.25 | 0.6 | 100 | 0.288 | 0.266 | 0.368 | 0.332 | 0.328 | 0.396 | -0.047 | -0.125 | -0.101 |
| Linear | 0.25 | 0.6 | 150 | 0.352 | 0.422 | 0.476 | 0.434 | 0.51 | 0.54 | -0.188 | 0.131 | -0.041 |
| Linear | 0.25 | 0.6 | 200 | 0.494 | 0.528 | 0.624 | 0.546 | 0.596 | 0.674 | -0.021 | -0.066 | -0.091 |
| Linear | 0.25 | 0.6 | 250 | 0.608 | 0.628 | 0.744 | 0.66 | 0.66 | 0.758 | 0.017 | -0.050 | -0.030 |
| Linear | 0.5 | 0 | 50 | 0.476 | 0.494 | 0.8 | 0.55 | 0.572 | 0.848 | -0.426 | -0.414 | -0.334 |
| Linear | 0.5 | 0 | 100 | 0.748 | 0.794 | 0.992 | 0.824 | 0.868 | 0.996 | -0.161 | -0.189 | -0.227 |
| Linear | 0.5 | 0 | 150 | 0.916 | 0.932 | 1 | 0.946 | 0.96 | 1 | 0.112 | 0.057 | 0.105 |
| Linear | 0.5 | 0 | 200 | 0.98 | 0.986 | 1 | 0.994 | 0.992 | 1 | 0.197 | -0.022 | 0.139 |
| Linear | 0.5 | 0 | 250 | 0.996 | 0.996 | 1 | 0.996 | 1 | 1 | 0.035 | 0.072 | 0.079 |
| Linear | 0.5 | 0.3 | 50 | 0.442 | 0.536 | 0.71 | 0.518 | 0.59 | 0.728 | -0.551 | -0.087 | -0.253 |
| Linear | 0.5 | 0.3 | 100 | 0.778 | 0.794 | 0.932 | 0.848 | 0.872 | 0.974 | -0.043 | -0.191 | -0.063 |
| Linear | 0.5 | 0.3 | 150 | 0.936 | 0.938 | 0.998 | 0.964 | 0.956 | 0.998 | 0.115 | -0.025 | 0.033 |
| Linear | 0.5 | 0.3 | 200 | 0.968 | 0.974 | 1 | 0.98 | 0.982 | 1 | 0.034 | -0.075 | 0.000 |
| Linear | 0.5 | 0.3 | 250 | 0.996 | 0.992 | 1 | 0.998 | 0.996 | 1 | 0.113 | -0.019 | 0.075 |
| Linear | 0.5 | 0.6 | 50 | 0.49 | 0.502 | 0.612 | 0.56 | 0.592 | 0.664 | -0.167 | -0.158 | -0.033 |
| Linear | 0.5 | 0.6 | 100 | 0.772 | 0.84 | 0.904 | 0.838 | 0.882 | 0.94 | -0.044 | 0.048 | 0.071 |
| Linear | 0.5 | 0.6 | 150 | 0.896 | 0.936 | 0.976 | 0.93 | 0.962 | 0.984 | 0.026 | -0.062 | 0.018 |
| Linear | 0.5 | 0.6 | 200 | 0.962 | 0.984 | 1 | 0.99 | 0.99 | 1 | -0.144 | -0.061 | -0.043 |
| Linear | 0.5 | 0.6 | 250 | 0.998 | 0.992 | 1 | 0.998 | 0.998 | 1 | -0.059 | -0.077 | -0.060 |
| Non-linear | 0 | 0 | 50 | 0.04 | 0.026 | 0.034 | 0.046 | 0.038 | 0.044 | -0.721 | -1.071 | -0.379 |
| Non-linear | 0 | 0 | 100 | 0.05 | 0.036 | 0.046 | 0.052 | 0.036 | 0.054 | -0.585 | -0.678 | -0.249 |
| Non-linear | 0 | 0 | 150 | 0.024 | 0.04 | 0.04 | 0.04 | 0.04 | 0.052 | -0.323 | -0.649 | -0.229 |
| Non-linear | 0 | 0 | 200 | 0.054 | 0.046 | 0.056 | 0.06 | 0.054 | 0.058 | -0.371 | -0.515 | -0.224 |
| Non-linear | 0 | 0 | 250 | 0.046 | 0.054 | 0.052 | 0.052 | 0.062 | 0.072 | -0.382 | -0.502 | -0.256 |
| Non-linear | 0 | 0.3 | 50 | 0.032 | 0.04 | 0.044 | 0.05 | 0.048 | 0.054 | -1.021 | -1.079 | -0.525 |
| Non-linear | 0 | 0.3 | 100 | 0.036 | 0.04 | 0.04 | 0.062 | 0.05 | 0.068 | -0.461 | -0.691 | -0.203 |
| Non-linear | 0 | 0.3 | 150 | 0.032 | 0.05 | 0.03 | 0.04 | 0.058 | 0.038 | -0.291 | -0.636 | -0.217 |
| Non-linear | 0 | 0.3 | 200 | 0.028 | 0.046 | 0.038 | 0.026 | 0.054 | 0.042 | -0.416 | -0.547 | -0.260 |
| Non-linear | 0 | 0.3 | 250 | 0.046 | 0.054 | 0.038 | 0.046 | 0.052 | 0.056 | -0.223 | -0.319 | -0.082 |
| Non-linear | 0 | 0.6 | 50 | 0.062 | 0.034 | 0.036 | 0.054 | 0.032 | 0.048 | -0.985 | -1.153 | -0.494 |
| Non-linear | 0 | 0.6 | 100 | 0.054 | 0.034 | 0.046 | 0.05 | 0.06 | 0.058 | -0.632 | -0.647 | -0.362 |
| Non-linear | 0 | 0.6 | 150 | 0.042 | 0.04 | 0.04 | 0.066 | 0.052 | 0.054 | -0.284 | -0.618 | -0.238 |
| Non-linear | 0 | 0.6 | 200 | 0.036 | 0.05 | 0.046 | 0.038 | 0.058 | 0.04 | -0.457 | -0.512 | -0.264 |
| Non-linear | 0 | 0.6 | 250 | 0.044 | 0.04 | 0.036 | 0.04 | 0.03 | 0.036 | -0.337 | -0.461 | -0.241 |
| Non-linear | 0.25 | 0 | 50 | 0.194 | 0.226 | 0.472 | 0.3 | 0.504 | 0.656 | 0.065 | 0.304 | 0.233 |
| Non-linear | 0.25 | 0 | 100 | 0.36 | 0.528 | 0.794 | 0.496 | 0.756 | 0.908 | 0.159 | 0.115 | 0.086 |
| Non-linear | 0.25 | 0 | 150 | 0.542 | 0.738 | 0.97 | 0.706 | 0.904 | 0.99 | 0.192 | 0.044 | 0.021 |
| Non-linear | 0.25 | 0 | 200 | 0.694 | 0.872 | 0.984 | 0.828 | 0.962 | 0.992 | 0.147 | 0.056 | 0.024 |
| Non-linear | 0.25 | 0 | 250 | 0.798 | 0.974 | 1 | 0.868 | 0.99 | 1 | 0.067 | 0.221 | 0.132 |
| Non-linear | 0.25 | 0.3 | 50 | 0.168 | 0.192 | 0.334 | 0.284 | 0.462 | 0.532 | 0.279 | 0.025 | 0.142 |
| Non-linear | 0.25 | 0.3 | 100 | 0.368 | 0.528 | 0.652 | 0.534 | 0.752 | 0.822 | 0.069 | 0.175 | 0.039 |
| Non-linear | 0.25 | 0.3 | 150 | 0.568 | 0.742 | 0.862 | 0.702 | 0.894 | 0.94 | 0.186 | 0.134 | 0.108 |
| Non-linear | 0.25 | 0.3 | 200 | 0.706 | 0.88 | 0.968 | 0.804 | 0.97 | 0.99 | 0.143 | 0.085 | 0.056 |
| Non-linear | 0.25 | 0.3 | 250 | 0.812 | 0.952 | 0.986 | 0.888 | 0.986 | 0.992 | 0.065 | 0.075 | 0.021 |
| Non-linear | 0.25 | 0.6 | 50 | 0.198 | 0.27 | 0.298 | 0.326 | 0.496 | 0.492 | 0.097 | 0.519 | 0.407 |
| Non-linear | 0.25 | 0.6 | 100 | 0.37 | 0.526 | 0.57 | 0.506 | 0.764 | 0.74 | 0.178 | 0.198 | 0.153 |
| Non-linear | 0.25 | 0.6 | 150 | 0.568 | 0.772 | 0.82 | 0.708 | 0.88 | 0.906 | 0.210 | 0.165 | 0.114 |
| Non-linear | 0.25 | 0.6 | 200 | 0.702 | 0.894 | 0.922 | 0.81 | 0.972 | 0.964 | 0.057 | 0.098 | 0.056 |
| Non-linear | 0.25 | 0.6 | 250 | 0.82 | 0.97 | 0.972 | 0.912 | 0.992 | 0.994 | 0.127 | 0.178 | 0.119 |
| Non-linear | 0.5 | 0 | 50 | 0.522 | 0.614 | 0.904 | 0.716 | 0.902 | 0.976 | -0.196 | 0.492 | -0.093 |
| Non-linear | 0.5 | 0 | 100 | 0.826 | 0.926 | 1 | 0.95 | 0.998 | 1 | 0.082 | 0.479 | 0.011 |
| Non-linear | 0.5 | 0 | 150 | 0.956 | 0.994 | 1 | 0.99 | 1 | 1 | 0.189 | 0.208 | 0.055 |
| Non-linear | 0.5 | 0 | 200 | 0.998 | 1 | 1 | 1 | 1 | 1 | 0.110 | 0.297 | 0.055 |
| Non-linear | 0.5 | 0 | 250 | 1 | 0.998 | 1 | 1 | 1 | 1 | 0.072 | 0.458 | 0.158 |
| Non-linear | 0.5 | 0.3 | 50 | 0.534 | 0.664 | 0.84 | 0.726 | 0.922 | 0.952 | 0.065 | 0.772 | 0.253 |
| Non-linear | 0.5 | 0.3 | 100 | 0.844 | 0.942 | 0.996 | 0.946 | 0.998 | 1 | 0.012 | 0.378 | 0.014 |
| Non-linear | 0.5 | 0.3 | 150 | 0.954 | 0.996 | 1 | 0.998 | 1 | 1 | 0.044 | 0.563 | 0.166 |
| Non-linear | 0.5 | 0.3 | 200 | 0.99 | 1 | 1 | 1 | 1 | 1 | 0.172 | 0.371 | 0.159 |
| Non-linear | 0.5 | 0.3 | 250 | 1 | 1 | 1 | 1 | 1 | 1 | 0.150 | 0.152 | 0.047 |
| Non-linear | 0.5 | 0.6 | 50 | 0.55 | 0.614 | 0.728 | 0.77 | 0.902 | 0.912 | 0.380 | 0.661 | 0.362 |
| Non-linear | 0.5 | 0.6 | 100 | 0.84 | 0.944 | 0.962 | 0.95 | 0.994 | 0.996 | 0.229 | 0.378 | 0.227 |
| Non-linear | 0.5 | 0.6 | 150 | 0.956 | 0.99 | 1 | 0.982 | 1 | 1 | -0.094 | 0.262 | -0.064 |
| Non-linear | 0.5 | 0.6 | 200 | 0.99 | 1 | 1 | 0.994 | 1 | 1 | 0.053 | 0.205 | 0.056 |
| Non-linear | 0.5 | 0.6 | 250 | 0.998 | 1 | 1 | 1 | 1 | 1 | 0.091 | 0.144 | 0.014 |

**Table A.1.** Null hypothesis rejection rates (type I error for zero effects; power for non-zero effects) and time savings bias across simulation scenarios for endpoint 1 TCT, endpoint 2 TCT, and gTCT.

| Trajectory shape | Linear | Linear | Linear | Linear | Non-linear | Non-linear | Non-linear | Non-linear |
| --- | --- | --- | --- | --- | --- | --- | --- | --- |
| Effect – time shrinking | 0 | 0 | 0.25 | 0.25 | 0 | 0 | 0.25 | 0.25 |
| Sample size per arm | 100 | 200 | 100 | 200 | 100 | 200 | 100 | 200 |
| Rejection rate – endpoint 1 | 0.069 | 0.050 | 0.257 | 0.525 | 0.040 | 0.030 | 0.257 | 0.624 |
| Rejection rate – endpoint 2 | 0.040 | 0.030 | 0.347 | 0.554 | 0.010 | 0.030 | 0.505 | 0.901 |
| Rejection rate – gTCT | 0.030 | 0.040 | 0.455 | 0.762 | 0.020 | 0.020 | 0.624 | 0.941 |
| Rejection rate – endpoint 1 – bootstrap | 0.030 | 0.069 | 0.347 | 0.624 | 0.030 | 0.020 | 0.287 | 0.673 |
| Rejection rate – endpoint 2 – bootstrap | 0.040 | 0.020 | 0.455 | 0.644 | 0.010 | 0.010 | 0.594 | 0.950 |
| Rejection rate – gTCT – bootstrap | 0.010 | 0.020 | 0.505 | 0.723 | 0.020 | 0.020 | 0.693 | 0.970 |
| SE – endpoint 1 | 3.226 | 2.307 | 3.437 | 2.326 | 2.861 | 1.965 | 2.753 | 1.872 |
| SE – endpoint 2 | 2.979 | 2.144 | 3.165 | 2.180 | 2.244 | 1.611 | 2.330 | 1.539 |
| SE – gTCT | 2.333 | 1.694 | 2.531 | 1.750 | 1.918 | 1.369 | 1.944 | 1.317 |
| SE – endpoint 1 – bootstrap | 3.453 | 2.311 | 3.157 | 2.115 | 3.161 | 2.039 | 2.647 | 1.738 |
| SE – endpoint 2 – bootstrap | 3.079 | 2.126 | 2.899 | 2.004 | 2.583 | 1.752 | 2.107 | 1.371 |
| SE – gTCT – bootstrap | 2.441 | 1.720 | 2.441 | 1.719 | 2.088 | 1.452 | 1.815 | 1.235 |

**Table A.2.** Null hypothesis rejection rates (type I error for zero effects; power for non-zero effects) and average standard errors across simulation scenarios for endpoint 1 TCT, endpoint 2 TCT, and gTCT compared between direct standard error construction and patient-wise bootstrap standard error.

| Trajectory shape | Effect – time shrinking – endpoint 1 | Effect – time shrinking – endpoint 2 | Correlation between endpoints | Sample size per arm | Rejection rate – TCT endpoint 1 | Rejection rate – TCT endpoint 2 | Rejection rate – gTCT |
| --- | --- | --- | --- | --- | --- | --- | --- |
| Linear | 0 | 0.25 | 0 | 50 | 0.042 | 0.15 | 0.116 |
| Linear | 0 | 0.25 | 0 | 100 | 0.04 | 0.264 | 0.202 |
| Linear | 0 | 0.25 | 0 | 150 | 0.026 | 0.398 | 0.236 |
| Linear | 0 | 0.25 | 0 | 200 | 0.04 | 0.576 | 0.356 |
| Linear | 0 | 0.25 | 0 | 250 | 0.06 | 0.61 | 0.38 |
| Linear | 0 | 0.25 | 0.3 | 50 | 0.042 | 0.194 | 0.12 |
| Linear | 0 | 0.25 | 0.3 | 100 | 0.036 | 0.286 | 0.146 |
| Linear | 0 | 0.25 | 0.3 | 150 | 0.052 | 0.406 | 0.196 |
| Linear | 0 | 0.25 | 0.3 | 200 | 0.032 | 0.572 | 0.276 |
| Linear | 0 | 0.25 | 0.3 | 250 | 0.032 | 0.666 | 0.33 |
| Linear | 0 | 0.25 | 0.6 | 50 | 0.054 | 0.174 | 0.096 |
| Linear | 0 | 0.25 | 0.6 | 100 | 0.034 | 0.25 | 0.136 |
| Linear | 0 | 0.25 | 0.6 | 150 | 0.026 | 0.396 | 0.184 |
| Linear | 0 | 0.25 | 0.6 | 200 | 0.04 | 0.556 | 0.264 |
| Linear | 0 | 0.25 | 0.6 | 250 | 0.032 | 0.642 | 0.298 |
| Linear | 0 | 0.5 | 0 | 50 | 0.04 | 0.504 | 0.342 |
| Linear | 0 | 0.5 | 0 | 100 | 0.034 | 0.8 | 0.532 |
| Linear | 0 | 0.5 | 0 | 150 | 0.05 | 0.92 | 0.664 |
| Linear | 0 | 0.5 | 0 | 200 | 0.034 | 0.988 | 0.798 |
| Linear | 0 | 0.5 | 0 | 250 | 0.052 | 0.996 | 0.888 |
| Linear | 0 | 0.5 | 0.3 | 50 | 0.036 | 0.536 | 0.302 |
| Linear | 0 | 0.5 | 0.3 | 100 | 0.032 | 0.764 | 0.434 |
| Linear | 0 | 0.5 | 0.3 | 150 | 0.046 | 0.954 | 0.58 |
| Linear | 0 | 0.5 | 0.3 | 200 | 0.028 | 0.972 | 0.658 |
| Linear | 0 | 0.5 | 0.3 | 250 | 0.036 | 0.994 | 0.756 |
| Linear | 0 | 0.5 | 0.6 | 50 | 0.04 | 0.502 | 0.286 |
| Linear | 0 | 0.5 | 0.6 | 100 | 0.032 | 0.798 | 0.424 |
| Linear | 0 | 0.5 | 0.6 | 150 | 0.048 | 0.948 | 0.542 |
| Linear | 0 | 0.5 | 0.6 | 200 | 0.032 | 0.988 | 0.614 |
| Linear | 0 | 0.5 | 0.6 | 250 | 0.03 | 0.994 | 0.706 |
| Non-linear | 0 | 0.25 | 0 | 50 | 0.054 | 0.248 | 0.156 |
| Non-linear | 0 | 0.25 | 0 | 100 | 0.036 | 0.544 | 0.338 |
| Non-linear | 0 | 0.25 | 0 | 150 | 0.038 | 0.748 | 0.492 |
| Non-linear | 0 | 0.25 | 0 | 200 | 0.032 | 0.896 | 0.668 |
| Non-linear | 0 | 0.25 | 0 | 250 | 0.03 | 0.962 | 0.808 |
| Non-linear | 0 | 0.25 | 0.3 | 50 | 0.04 | 0.242 | 0.142 |
| Non-linear | 0 | 0.25 | 0.3 | 100 | 0.06 | 0.538 | 0.288 |
| Non-linear | 0 | 0.25 | 0.3 | 150 | 0.026 | 0.77 | 0.472 |
| Non-linear | 0 | 0.25 | 0.3 | 200 | 0.038 | 0.91 | 0.63 |
| Non-linear | 0 | 0.25 | 0.3 | 250 | 0.03 | 0.96 | 0.732 |
| Non-linear | 0 | 0.25 | 0.6 | 50 | 0.042 | 0.218 | 0.112 |
| Non-linear | 0 | 0.25 | 0.6 | 100 | 0.042 | 0.528 | 0.304 |
| Non-linear | 0 | 0.25 | 0.6 | 150 | 0.036 | 0.788 | 0.474 |
| Non-linear | 0 | 0.25 | 0.6 | 200 | 0.054 | 0.91 | 0.62 |
| Non-linear | 0 | 0.25 | 0.6 | 250 | 0.054 | 0.966 | 0.792 |
| Non-linear | 0 | 0.5 | 0 | 50 | 0.02 | 0.638 | 0.364 |
| Non-linear | 0 | 0.5 | 0 | 100 | 0.04 | 0.952 | 0.602 |
| Non-linear | 0 | 0.5 | 0 | 150 | 0.038 | 0.988 | 0.766 |
| Non-linear | 0 | 0.5 | 0 | 200 | 0.026 | 0.998 | 0.866 |
| Non-linear | 0 | 0.5 | 0 | 250 | 0.044 | 1 | 0.922 |
| Non-linear | 0 | 0.5 | 0.3 | 50 | 0.034 | 0.57 | 0.258 |
| Non-linear | 0 | 0.5 | 0.3 | 100 | 0.03 | 0.944 | 0.566 |
| Non-linear | 0 | 0.5 | 0.3 | 150 | 0.03 | 0.996 | 0.662 |
| Non-linear | 0 | 0.5 | 0.3 | 200 | 0.032 | 0.996 | 0.774 |
| Non-linear | 0 | 0.5 | 0.3 | 250 | 0.048 | 1 | 0.816 |
| Non-linear | 0 | 0.5 | 0.6 | 50 | 0.05 | 0.652 | 0.304 |
| Non-linear | 0 | 0.5 | 0.6 | 100 | 0.04 | 0.926 | 0.452 |
| Non-linear | 0 | 0.5 | 0.6 | 150 | 0.04 | 0.998 | 0.574 |
| Non-linear | 0 | 0.5 | 0.6 | 200 | 0.062 | 1 | 0.666 |
| Non-linear | 0 | 0.5 | 0.6 | 250 | 0.032 | 1 | 0.732 |

**Table A.3.** Null hypothesis rejection rates (type I error for zero effects; power for non-zero effects) for mixed null and non-null treatment effect simulation scenarios for endpoint 1 TCT, endpoint 2 TCT, and gTCT.

## Additional Results for All Patients and Mild Patients Only with Comparisons Of 2 Mg AD04 Against 1 Mg AD04 And All Other Study Arms Combined


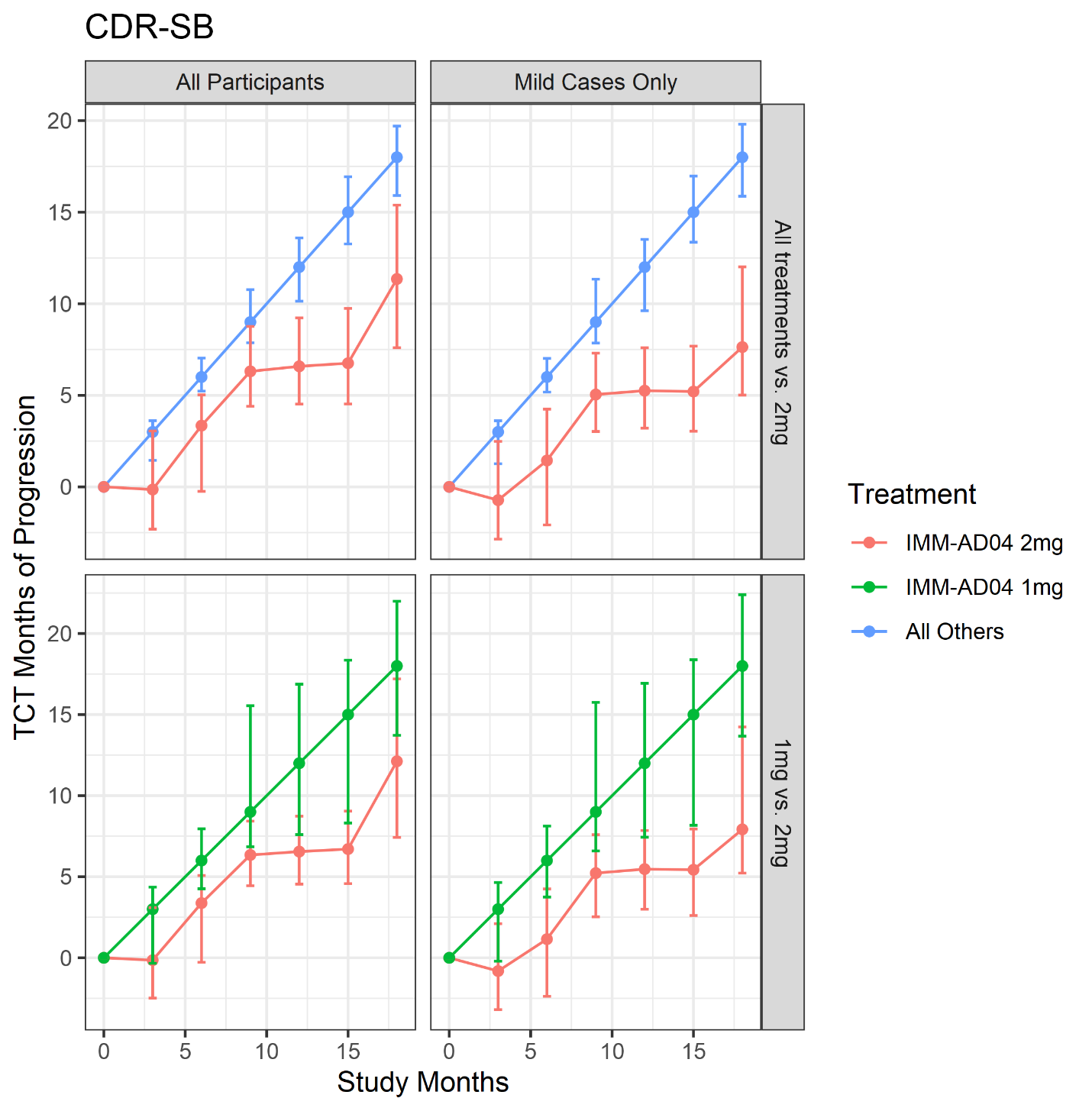


Figure A.1A. Mean changes over time in the active vs control group on CDR-sb


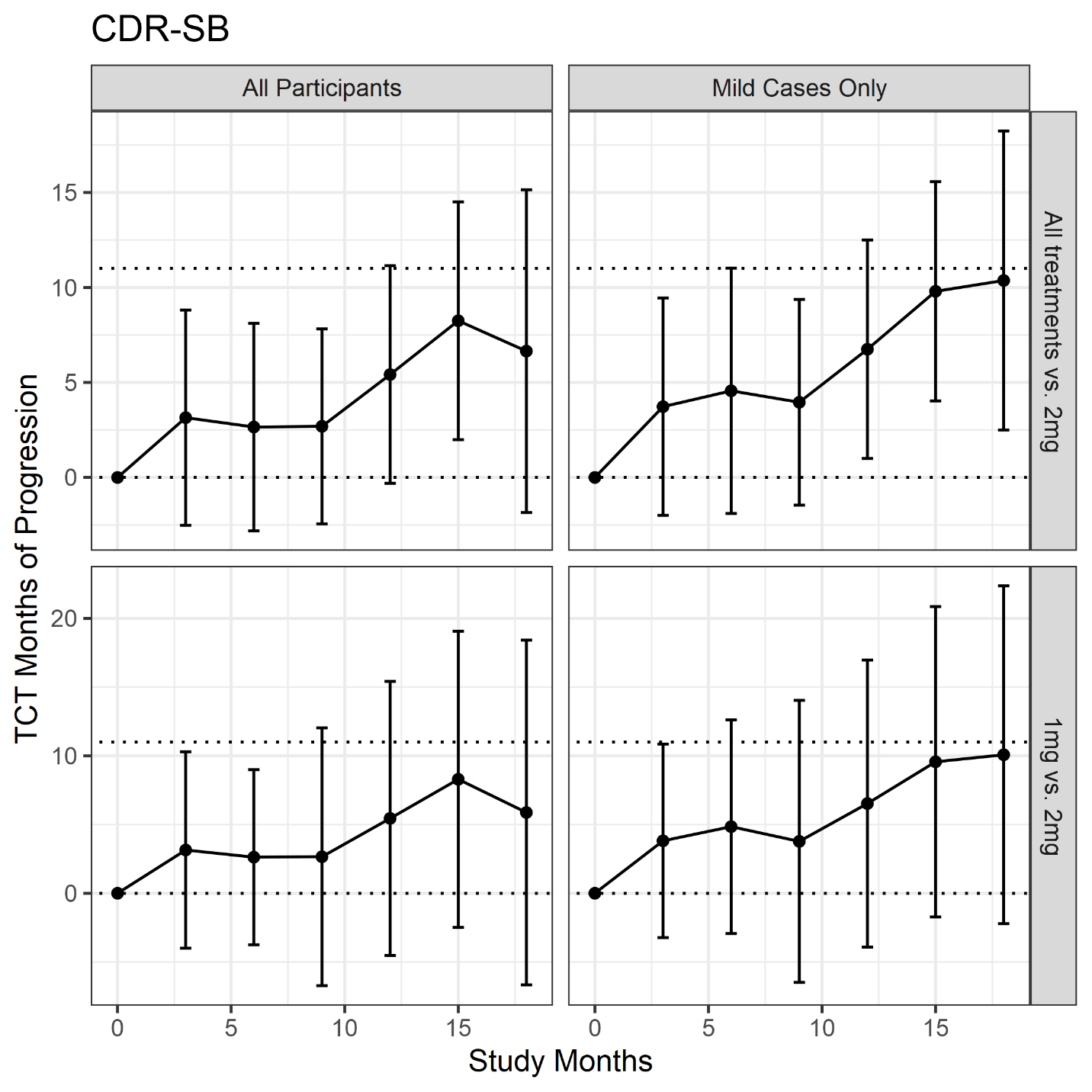


Figure A.1B. Time saved with active treatment (i.e., the horizontal difference between study arms) for CDR-sb.


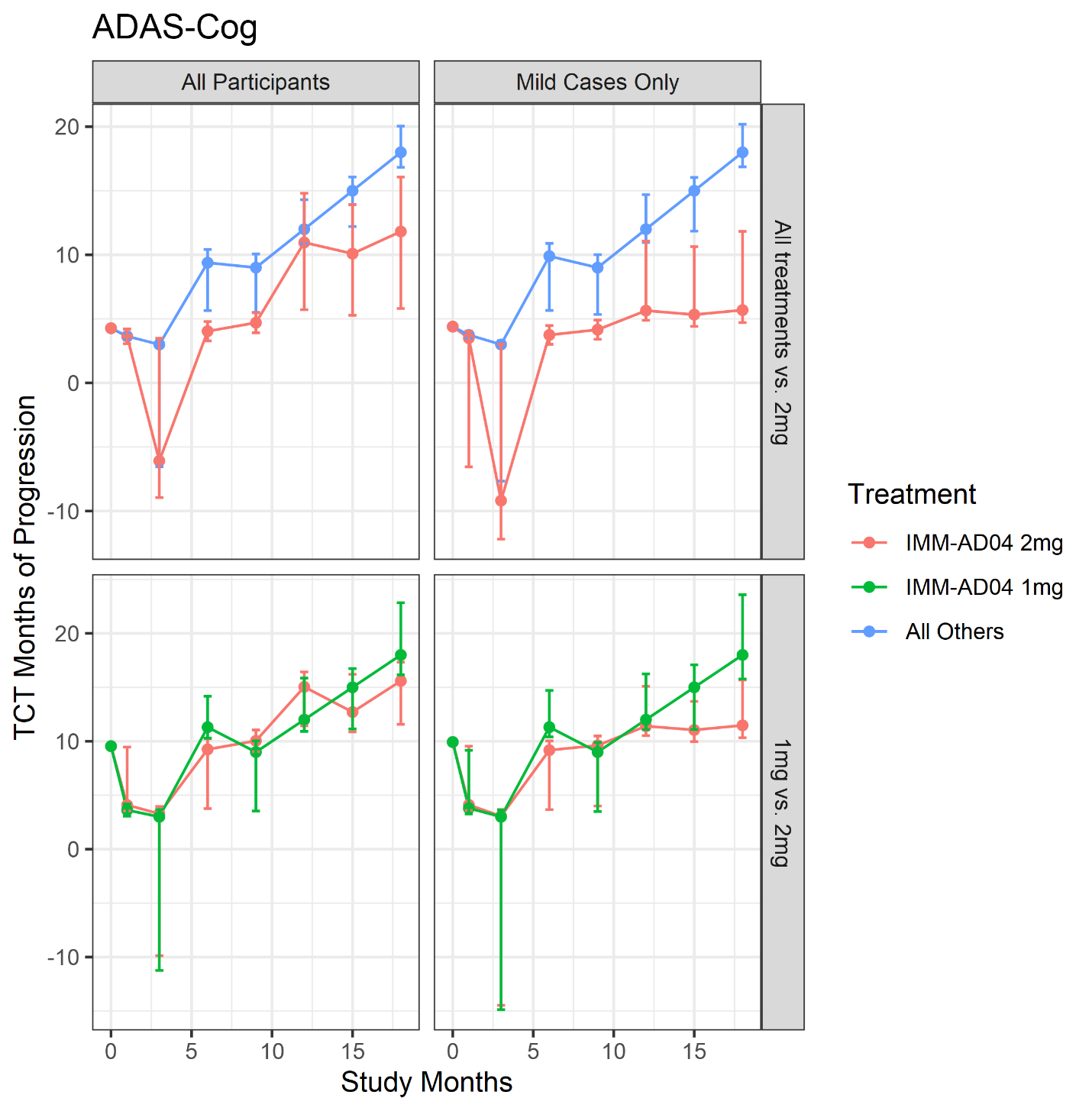


Figure A.2A. Mean changes over time in the active vs control group on aADAS-Cog


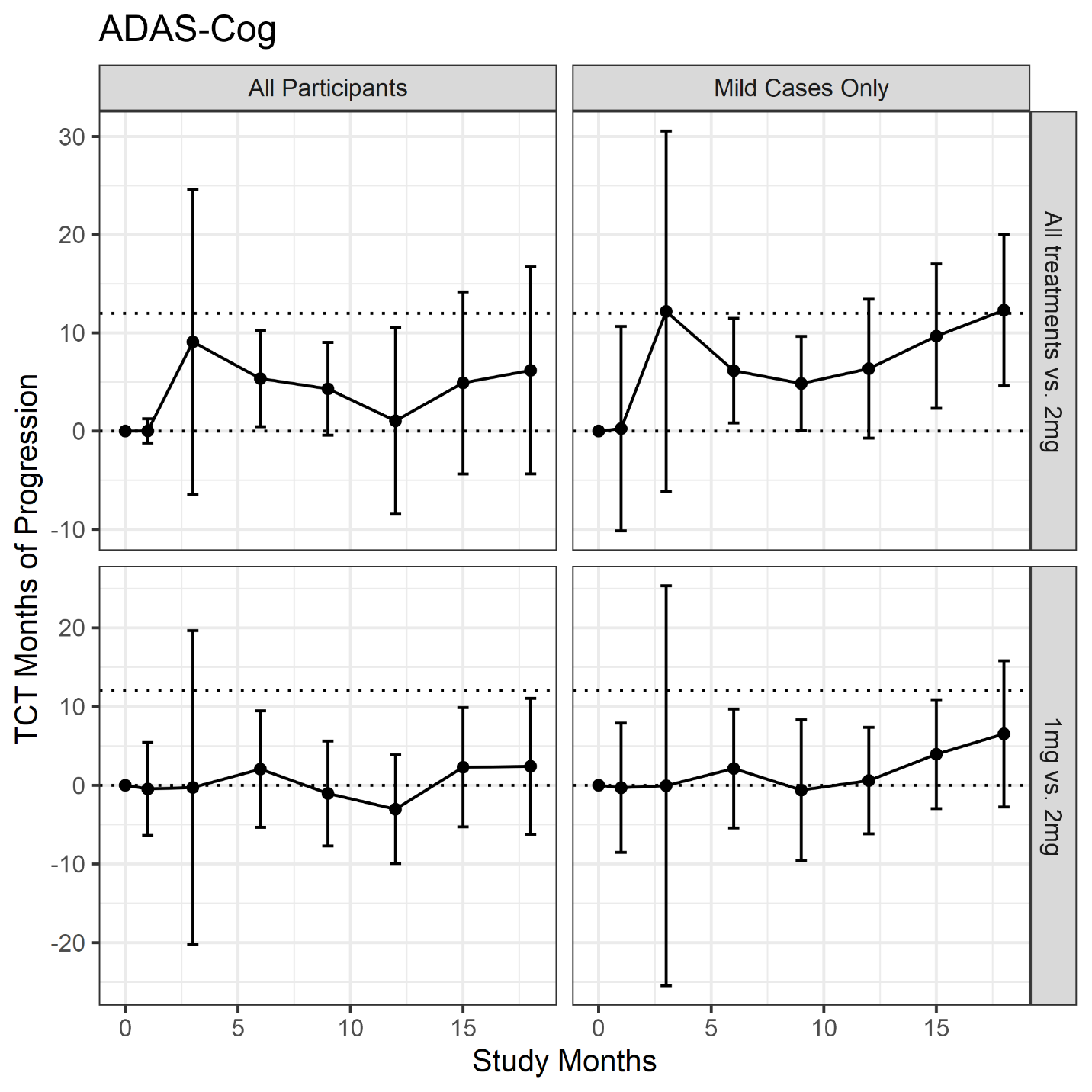


Figure A.2B. Time saved with active treatment (i.e., the horizontal difference between study arms) for aADAS-Cog.


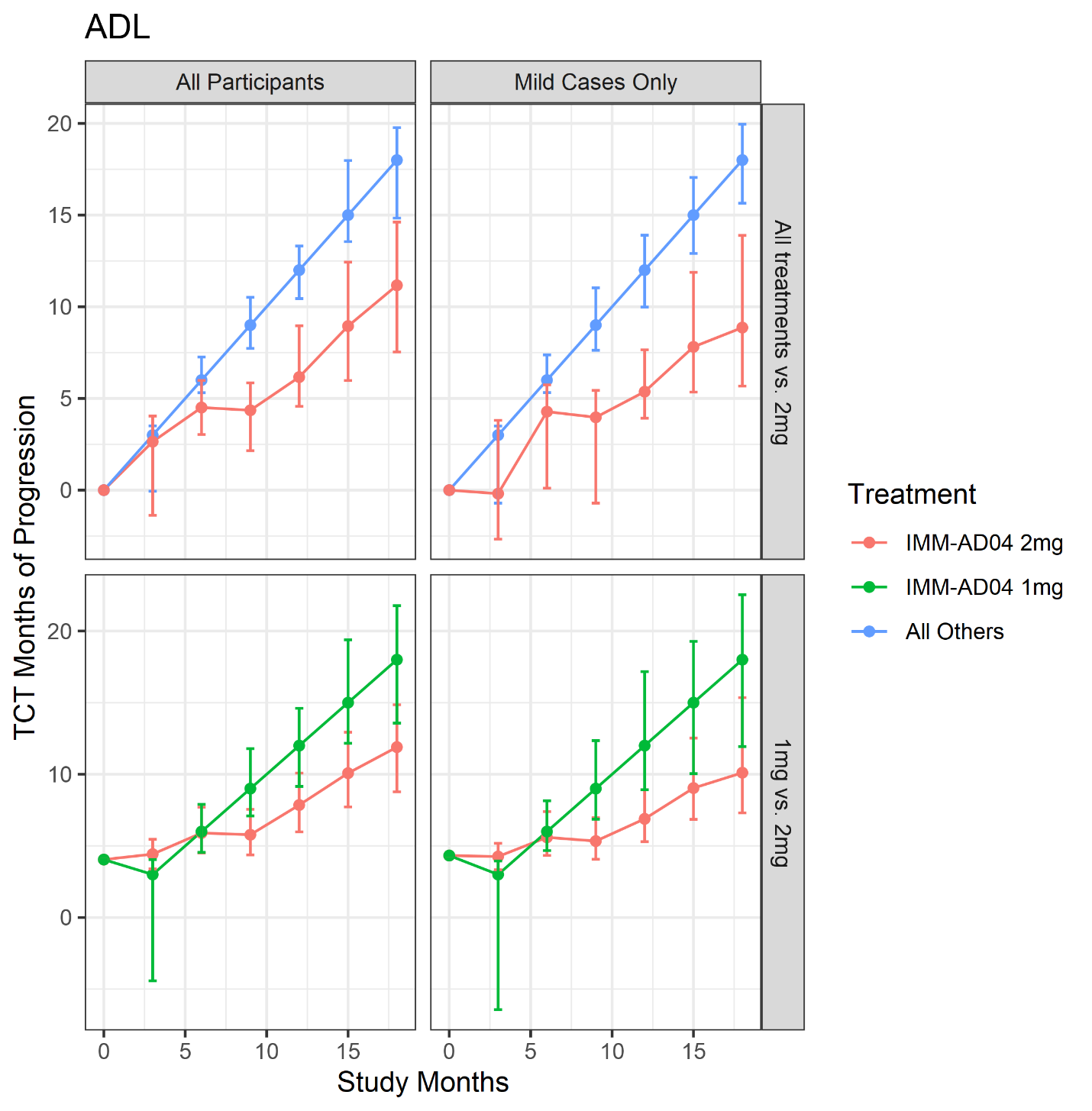


Figure A.3A. Mean changes over time in the active vs control group on aADL


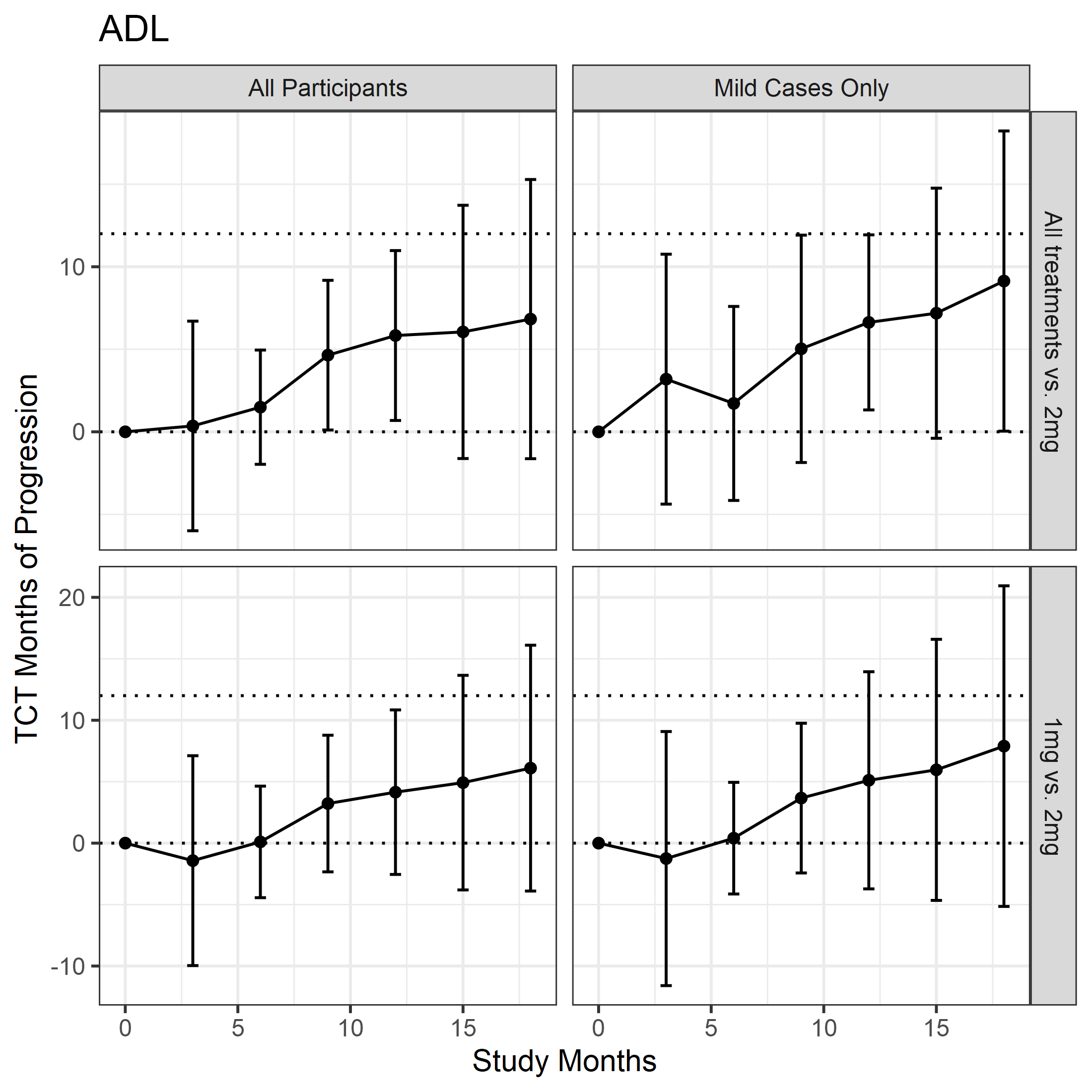


Figure A.3B. Time saved with active treatment (i.e., the horizontal difference between study arms) for aADL.

| Cohort | Comparison arm | Endpoint | Mean change (SE) to month 18 | | | Mean difference (95% CI) | Time saved (months – 95% CI) |
| --- | --- | --- | --- | --- | --- | --- | --- |
|  |  |  | 2mg AD04 arm | Comparison arm | |  |  |
| All patients | All other study arms combined | aADAS | 3.04 (1.68) | 6.69 (0.83) | | -3.65 (-7.32, 0.02) | 8.72 (-1.81, 19.25) |
| All patients | All other study arms combined | aADL | -8.32 (2.74) | -14.19 (1.38) | | 5.87 (-0.15, 11.88) | 9.52 (1.19, 17.86) |
| All patients | All other study arms combined | CDR-sb | 1.33 (0.38) | 1.95 (0.18) | | -0.61 (-1.45, 0.22) | 6.65 (-1.84, 15.14) |
| All patients | All other study arms combined | Composite | 2.43 (1.81) | 6.27 (0.90) | | -3.85 (-7.82, 0.12) | 11.11 (2.22, 20.01) |
| All patients | All other study arms combined | ADAS-cog | 3.49 (1.38) | 5.99 (0.68) | | -2.51 (-5.52, 0.51) | 6.18 (-4.36, 16.72) |
| All patients | All other study arms combined | ADCS-ADL | -5.89 (1.72) | -8.57 (0.84) | | 2.68 (-1.08, 6.44) | 6.83 (-1.63, 15.29) |
| All patients | All other study arms combined | gTCT1: aADAS, aADL, and CDR-sb | | | | | 8.34 (1.18, 15.50) |
| All patients | All other study arms combined | gTCT2: CDR-sb and Composite | | | | | 8.70 (1.32, 16.07) |
| All patients | All other study arms combined | gTCT3: ADAS-cog, ADCS-ADL, and CDR-sb | | | | | 6.63 (-0.70, 13.96) |
| All patients | 1mg AD04 | aADAS | 3.10 (1.68) | 6.17 (1.82) | | -3.06 (-7.92, 1.80) | 2.10 (-7.32, 11.52) |
| All patients | 1mg AD04 | aADL | -8.34 (2.76) | -14.71 (3.04) | | 6.37 (-1.67, 14.41) | 6.65 (-3.43, 16.74) |
| All patients | 1mg AD04 | CDR-sb | 1.34 (0.38) | 1.81 (0.40) | | -0.48 (-1.57, 0.61) | 5.88 (-6.67, 18.43) |
| All patients | 1mg AD04 | Composite | 2.44 (1.82) | 6.43 (1.98) | | -3.99 (-9.27, 1.28) | 8.41 (-4.08, 20.89) |
| All patients | 1mg AD04 | ADAS-cog | 3.53 (1.39) | 5.45 (1.46) | | -1.91 (-5.86, 2.04) | 2.41 (-6.23, 11.05) |
| All patients | 1mg AD04 | ADCS-ADL | -5.90 (1.73) | -8.70 (1.82) | | 2.80 (-2.12, 7.72) | 6.10 (-3.90, 16.11) |
| All patients | 1mg AD04 | gTCT1: aADAS, aADL, and CDR-sb | | | | | 4.17 (-3.85, 12.19) |
| All patients | 1mg AD04 | gTCT2: CDR-sb and Composite | | | | | 7.15 (-3.48, 17.79) |
| All patients | 1mg AD04 | gTCT3: ADAS-cog, ADCS-ADL, and CDR-sb | | | | | 3.80 (-4.06, 11.67) |
| Mild patients | All other study arms combined | aADAS | 1.26 (1.69) | | 6.57 (0.85) | -5.31 (-9.01, -1.61) | 12.56 (6.62, 18.50) |
| Mild patients | All other study arms combined | aADL | -6.71 (2.80) | | -13.22 (1.40) | 6.51 (0.38, 12.64) | 11.24 (3.10, 19.37) |
| Mild patients | All other study arms combined | CDR-sb | 0.89 (0.38) | | 1.84 (0.18) | -0.95 (-1.79, -0.12) | 10.37 (2.50, 18.24) |
| Mild patients | All other study arms combined | Composite | 1.33 (1.83) | | 6.21 (0.91) | -4.88 (-8.89, -0.87) | 12.42 (6.55, 18.29) |
| Mild patients | All other study arms combined | ADAS-cog | 1.83 (1.38) | | 5.67 (0.69) | -3.84 (-6.87, -0.82) | 12.31 (4.61, 20.01) |
| Mild patients | All other study arms combined | ADCS-ADL | -4.62 (1.75) | | -7.85 (0.85) | 3.23 (-0.58, 7.03) | 9.13 (0.04, 18.23) |
| Mild patients | All other study arms combined | gTCT1: aADAS, aADL, and CDR-sb | | | | | 11.95 (6.56, 17.34) |
| Mild patients | All other study arms combined | gTCT2: CDR-sb and Composite | | | | | 11.86 (6.38, 17.35) |
| Mild patients | All other study arms combined | gTCT3: ADAS-cog, ADCS-ADL, and CDR-sb | | | | | 11.08 (4.61, 17.55) |
| Mild patients | 1mg AD04 | aADAS | 1.30 (1.69) | | 5.77 (1.85) | -4.47 (-9.38, 0.45) | 7.85 (-4.73, 20.43) |
| Mild patients | 1mg AD04 | aADL | -6.70 (2.81) | | -12.58 (3.12) | 5.89 (-2.35, 14.12) | 8.13 (-3.80, 20.06) |
| Mild patients | 1mg AD04 | CDR-sb | 0.89 (0.38) | | 1.66 (0.40) | -0.76 (-1.85, 0.33) | 10.08 (-2.22, 22.37) |
| Mild patients | 1mg AD04 | Composite | 1.31 (1.84) | | 5.51 (2.01) | -4.20 (-9.54, 1.14) | 9.77 (-0.74, 20.27) |
| Mild patients | 1mg AD04 | ADAS-cog | 1.86 (1.38) | | 4.78 (1.48) | -2.92 (-6.89, 1.06) | 6.53 (-2.76, 15.81) |
| Mild patients | 1mg AD04 | ADCS-ADL | -4.63 (1.75) | | -7.38 (1.86) | 2.74 (-2.26, 7.75) | 7.89 (-5.15, 20.93) |
| Mild patients | 1mg AD04 | gTCT1: aADAS, aADL, and CDR-sb | | | | | 8.55 (-1.13, 18.23) |
| Mild patients | 1mg AD04 | gTCT2: CDR-sb and Composite | | | | | 9.88 (0.48, 19.29) |
| Mild patients | 1mg AD04 | gTCT3: ADAS-cog, ADCS-ADL, and CDR-sb | | | | | 7.40 (-1.24, 16.04) |

**Table A.4.** Summary of mean changes from baseline to 18 months and time saved results for the 2mg AD04 arm as compared with all other study arms combined or the 1mg AD04 arm in the overall patient cohort or in patients with mild Alzheimer’s disease (Baseline MMSE >=20).

| Cohort | Comparison arm | Endpoint | Mean change (SE) to month 12 | | | Mean difference (95% CI) | Time saved (months – 95% CI) |
| --- | --- | --- | --- | --- | --- | --- | --- |
|  |  |  | 2mg AD04 arm | Comparison arm | |  |  |
| All patients | All other study arms combined | aADAS | 3.09 (1.39) | 4.43 (0.65) | | -1.35 (-4.35, 1.66) | 5.93 (-5.00, 16.86) |
| All patients | All other study arms combined | aADL | -5.27 (2.34) | -10.35 (1.12) | | 5.08 (-0.00, 10.16) | 6.63 (1.27, 12.00) |
| All patients | All other study arms combined | CDR-sb | 0.82 (0.34) | 1.39 (0.16) | | -0.57 (-1.30, 0.16) | 5.42 (-0.31, 11.14) |
| All patients | All other study arms combined | Composite | 2.51 (1.50) | 4.01 (0.72) | | -1.50 (-4.75, 1.75) | 4.94 (-4.13, 14.01) |
| All patients | All other study arms combined | ADAS-cog | 3.09 (1.11) | 3.57 (0.52) | | -0.48 (-2.87, 1.91) | 1.04 (-8.47, 10.55) |
| All patients | All other study arms combined | ADCS-ADL | -3.45 (1.47) | -6.26 (0.68) | | 2.81 (-0.37, 5.99) | 5.83 (0.69, 10.98) |
| All patients | All other study arms combined | gTCT1: aADAS, aADL, and CDR-sb | | | | | 6.11 (1.28, 10.93) |
| All patients | All other study arms combined | gTCT2: CDR-sb and Composite | | | | | 5.34 (-0.21, 10.88) |
| All patients | All other study arms combined | gTCT3: ADAS-cog, ADCS-ADL, and CDR-sb | | | | | 5.53 (0.80, 10.26) |
| All patients | 1mg AD04 | aADAS | 3.07 (1.39) | 3.38 (1.46) | | -0.31 (-4.26, 3.64) | 0.21 (-6.49, 6.92) |
| All patients | 1mg AD04 | aADL | -5.22 (2.34) | -8.83 (2.56) | | 3.60 (-3.20, 10.41) | 6.04 (-2.05, 14.14) |
| All patients | 1mg AD04 | CDR-sb | 0.82 (0.34) | 1.33 (0.35) | | -0.52 (-1.48, 0.45) | 5.45 (-4.53, 15.42) |
| All patients | 1mg AD04 | Composite | 2.51 (1.50) | 4.61 (1.65) | | -2.09 (-6.46, 2.27) | 6.02 (-3.30, 15.34) |
| All patients | 1mg AD04 | ADAS-cog | 3.10 (1.10) | 2.60 (1.15) | | 0.50 (-2.62, 3.62) | -3.04 (-9.93, 3.85) |
| All patients | 1mg AD04 | ADCS-ADL | -3.45 (1.47) | -5.95 (1.53) | | 2.50 (-1.66, 6.66) | 4.15 (-2.55, 10.84) |
| All patients | 1mg AD04 | gTCT1: aADAS, aADL, and CDR-sb | | | | | 2.39 (-3.54, 8.33) |
| All patients | 1mg AD04 | gTCT2: CDR-sb and Composite | | | | | 5.77 (-2.28, 13.81) |
| All patients | 1mg AD04 | gTCT3: ADAS-cog, ADCS-ADL, and CDR-sb | | | | | 0.72 (-4.91, 6.35) |
| Mild patients | All other study arms combined | aADAS | 1.66 (1.36) | | 4.24 (0.64) | -2.58 (-5.52, 0.36) | 9.80 (2.75, 16.85) |
| Mild patients | All other study arms combined | aADL | -4.24 (2.31) | | -9.44 (1.11) | 5.20 (0.19, 10.21) | 7.17 (0.38, 13.95) |
| Mild patients | All other study arms combined | CDR-sb | 0.55 (0.34) | | 1.27 (0.16) | -0.73 (-1.46, 0.01) | 6.75 (1.00, 12.49) |
| Mild patients | All other study arms combined | Composite | 1.10 (1.42) | | 4.20 (0.68) | -3.10 (-6.18, -0.02) | 6.57 (0.01, 13.13) |
| Mild patients | All other study arms combined | ADAS-cog | 1.77 (1.07) | | 3.29 (0.50) | -1.52 (-3.84, 0.80) | 6.36 (-0.72, 13.43) |
| Mild patients | All other study arms combined | ADCS-ADL | -2.57 (1.45) | | -5.70 (0.68) | 3.13 (-0.01, 6.27) | 6.63 (1.33, 11.93) |
| Mild patients | All other study arms combined | gTCT1: aADAS, aADL, and CDR-sb | | | | | 7.73 (2.73, 12.72) |
| Mild patients | All other study arms combined | gTCT2: CDR-sb and Composite | | | | | 6.68 (1.58, 11.77) |
| Mild patients | All other study arms combined | gTCT3: ADAS-cog, ADCS-ADL, and CDR-sb | | | | | 6.61 (1.99, 11.23) |
| Mild patients | 1mg AD04 | aADAS | 1.65 (1.35) | | 3.50 (1.43) | -1.84 (-5.70, 2.01) | 1.32 (-5.55, 8.19) |
| Mild patients | 1mg AD04 | aADL | -4.22 (2.31) | | -8.24 (2.55) | 4.02 (-2.73, 10.76) | 6.48 (-3.14, 16.10) |
| Mild patients | 1mg AD04 | CDR-sb | 0.54 (0.34) | | 1.18 (0.35) | -0.64 (-1.60, 0.33) | 6.53 (-3.92, 16.98) |
| Mild patients | 1mg AD04 | Composite | 1.12 (1.42) | | 4.59 (1.57) | -3.47 (-7.62, 0.68) | 9.64 (-0.92, 20.19) |
| Mild patients | 1mg AD04 | ADAS-cog | 1.78 (1.07) | | 2.50 (1.11) | -0.72 (-3.74, 2.30) | 0.60 (-6.16, 7.36) |
| Mild patients | 1mg AD04 | ADCS-ADL | -2.58 (1.46) | | -5.55 (1.51) | 2.97 (-1.15, 7.09) | 5.11 (-3.72, 13.95) |
| Mild patients | 1mg AD04 | gTCT1: aADAS, aADL, and CDR-sb | | | | | 2.91 (-3.41, 9.22) |
| Mild patients | 1mg AD04 | gTCT2: CDR-sb and Composite | | | | | 8.06 (-0.73, 16.84) |
| Mild patients | 1mg AD04 | gTCT3: ADAS-cog, ADCS-ADL, and CDR-sb | | | | | 2.13 (-4.02, 8.28) |

**Table A.5.** Summary of mean changes from baseline to 12 months and time saved results for the 2mg AD04 arm as compared with all other study arms combined or the 1mg AD04 arm in the overall patient cohort or in patients with mild Alzheimer’s disease (Baseline MMSE >=20).

| Cohort | Comparison arm | Endpoint | Mean change (SE) to month 6 | | | Mean difference (95% CI) | Time saved (months – 95% CI) |
| --- | --- | --- | --- | --- | --- | --- | --- |
|  |  |  | 2mg AD04 arm | Comparison arm | |  |  |
| All patients | All other study arms combined | aADAS | -1.12 (1.33) | 2.48 (0.60) | | -3.60 (-6.46, -0.74) | 1.95 (-1.94, 5.83) |
| All patients | All other study arms combined | aADL | -2.65 (2.16) | -6.25 (1.04) | | 3.60 (-1.10, 8.30) | 2.31 (-2.16, 6.78) |
| All patients | All other study arms combined | CDR-sb | 0.27 (0.30) | 0.74 (0.14) | | -0.47 (-1.11, 0.17) | 2.66 (-2.80, 8.11) |
| All patients | All other study arms combined | Composite | -1.46 (1.47) | 2.00 (0.70) | | -3.46 (-6.66, -0.27) | 2.33 (-10.62, 15.28) |
| All patients | All other study arms combined | ADAS-cog | -0.32 (1.03) | 2.37 (0.48) | | -2.69 (-4.91, -0.46) | 5.35 (0.45, 10.25) |
| All patients | All other study arms combined | ADCS-ADL | -1.92 (1.43) | -3.36 (0.66) | | 1.44 (-1.64, 4.52) | 1.49 (-1.97, 4.95) |
| All patients | All other study arms combined | gTCT1: aADAS, aADL, and CDR-sb | | | | | 2.18 (-1.05, 5.41) |
| All patients | All other study arms combined | gTCT2: CDR-sb and Composite | | | | | 2.63 (-2.72, 7.98) |
| All patients | All other study arms combined | gTCT3: ADAS-cog, ADCS-ADL, and CDR-sb | | | | | 2.50 (-0.63, 5.62) |
| All patients | 1mg AD04 | aADAS | -1.14 (1.34) | 1.41 (1.39) | | -2.55 (-6.33, 1.23) | 5.31 (-4.97, 15.59) |
| All patients | 1mg AD04 | aADL | -2.62 (2.17) | -5.30 (2.34) | | 2.68 (-3.58, 8.94) | 1.50 (-4.59, 7.59) |
| All patients | 1mg AD04 | CDR-sb | 0.27 (0.30) | 0.73 (0.30) | | -0.46 (-1.29, 0.38) | 2.63 (-3.75, 9.00) |
| All patients | 1mg AD04 | Composite | -1.49 (1.48) | 1.56 (1.60) | | -3.06 (-7.33, 1.21) | 2.04 (-12.01, 16.10) |
| All patients | 1mg AD04 | ADAS-cog | -0.31 (1.04) | 1.86 (1.08) | | -2.17 (-5.10, 0.75) | 2.05 (-5.35, 9.45) |
| All patients | 1mg AD04 | ADCS-ADL | -1.90 (1.43) | -2.00 (1.49) | | 0.10 (-3.94, 4.14) | 0.10 (-4.44, 4.64) |
| All patients | 1mg AD04 | gTCT1: aADAS, aADL, and CDR-sb | | | | | 2.26 (-2.75, 7.26) |
| All patients | 1mg AD04 | gTCT2: CDR-sb and Composite | | | | | 2.57 (-3.64, 8.77) |
| All patients | 1mg AD04 | gTCT3: ADAS-cog, ADCS-ADL, and CDR-sb | | | | | 0.94 (-3.17, 5.04) |
| Mild patients | All other study arms combined | aADAS | -1.91 (1.33) | | 2.32 (0.61) | -4.22 (-7.08, -1.36) | 2.24 (-1.86, 6.33) |
| Mild patients | All other study arms combined | aADL | -1.76 (2.21) | | -6.12 (1.06) | 4.36 (-0.44, 9.17) | 2.71 (-2.84, 8.25) |
| Mild patients | All other study arms combined | CDR-sb | 0.08 (0.30) | | 0.67 (0.14) | -0.59 (-1.23, 0.05) | 4.56 (-1.89, 11.01) |
| Mild patients | All other study arms combined | Composite | -2.20 (1.51) | | 1.97 (0.72) | -4.17 (-7.45, -0.89) | 2.73 (-12.14, 17.60) |
| Mild patients | All other study arms combined | ADAS-cog | -0.92 (1.04) | | 2.27 (0.48) | -3.20 (-5.44, -0.95) | 6.15 (0.83, 11.48) |
| Mild patients | All other study arms combined | ADCS-ADL | -1.47 (1.47) | | -3.20 (0.68) | 1.73 (-1.45, 4.90) | 1.72 (-4.16, 7.60) |
| Mild patients | All other study arms combined | gTCT1: aADAS, aADL, and CDR-sb | | | | | 2.71 (-0.91, 6.32) |
| Mild patients | All other study arms combined | gTCT2: CDR-sb and Composite | | | | | 4.39 (-1.91, 10.69) |
| Mild patients | All other study arms combined | gTCT3: ADAS-cog, ADCS-ADL, and CDR-sb | | | | | 4.49 (0.29, 8.70) |
| Mild patients | 1mg AD04 | aADAS | -1.92 (1.33) | | 1.03 (1.40) | -2.95 (-6.74, 0.83) | 5.36 (-4.80, 15.53) |
| Mild patients | 1mg AD04 | aADL | -1.73 (2.21) | | -5.19 (2.42) | 3.45 (-2.97, 9.88) | 1.71 (-4.85, 8.27) |
| Mild patients | 1mg AD04 | CDR-sb | 0.08 (0.30) | | 0.61 (0.31) | -0.54 (-1.37, 0.30) | 4.85 (-2.93, 12.63) |
| Mild patients | 1mg AD04 | Composite | -2.24 (1.52) | | 0.90 (1.66) | -3.14 (-7.54, 1.26) | 2.08 (-15.50, 19.65) |
| Mild patients | 1mg AD04 | ADAS-cog | -0.91 (1.04) | | 1.67 (1.09) | -2.57 (-5.54, 0.39) | 2.13 (-5.41, 9.68) |
| Mild patients | 1mg AD04 | ADCS-ADL | -1.48 (1.47) | | -1.95 (1.54) | 0.47 (-3.71, 4.64) | 0.40 (-4.14, 4.95) |
| Mild patients | 1mg AD04 | gTCT1: aADAS, aADL, and CDR-sb | | | | | 3.23 (-2.38, 8.83) |
| Mild patients | 1mg AD04 | gTCT2: CDR-sb and Composite | | | | | 4.58 (-3.01, 12.17) |
| Mild patients | 1mg AD04 | gTCT3: ADAS-cog, ADCS-ADL, and CDR-sb | | | | | 1.26 (-3.00, 5.51) |

**Table A.6.** Summary of mean changes from baseline to 6 months and time saved results for the 2mg AD04 arm as compared with all other study arms combined or the 1mg AD04 arm in the overall patient cohort or in patients with mild Alzheimer’s disease (Baseline MMSE >=20).

| 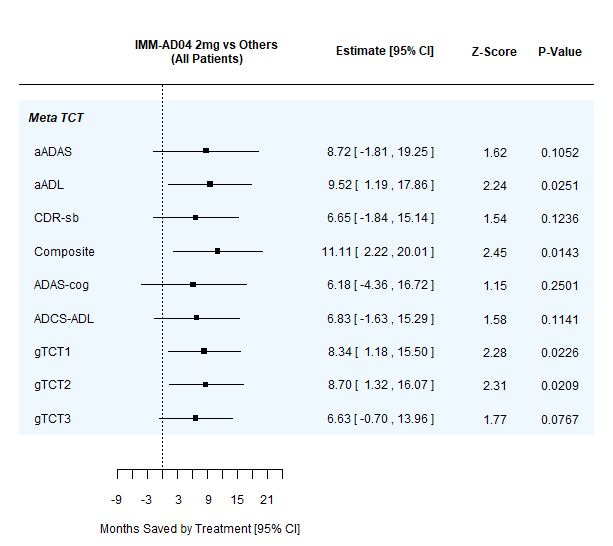 | 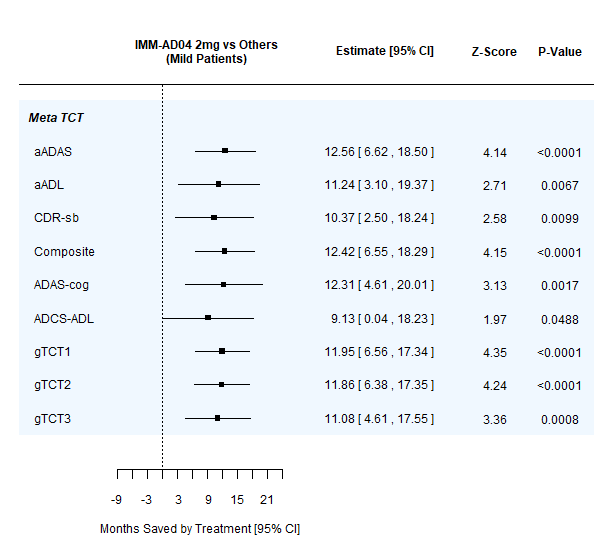 |
| --- | --- |
| 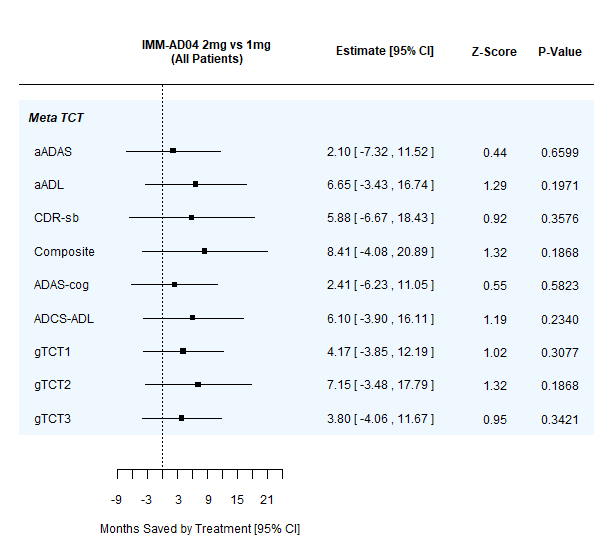 | 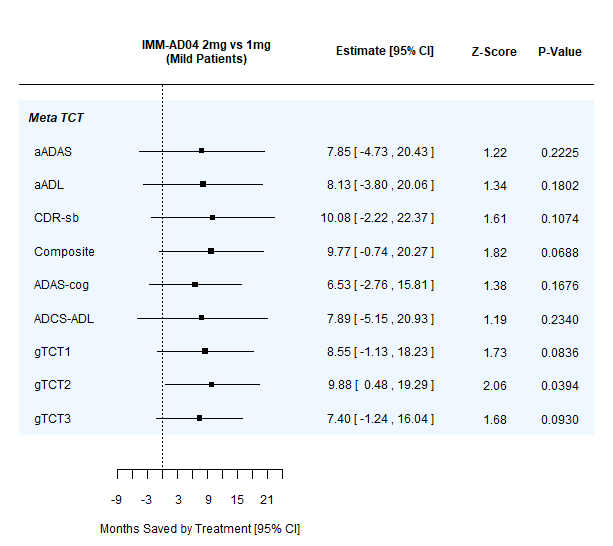 |

**Table A.7.** Forest plots summarizing time savings analyses at 18 months; left panels show full patient cohort, right panels show mild Alzheimer’s subgroup (baseline MMSE >=20); upper panels show 2mg AD04 vs. other treatment arms combined, lower panels show 2mg AD04 vs. 1mg AD04.

**References**

1. Ver Hoef, J.M., 2012. Who invented the delta method? *The American Statistician*, *66*(2), pp.124-127.
2. Nocedal, J. and Wright, S.J. eds., 1999. *Numerical optimization*. New York, NY: Springer New York.
3. B. A. Turlach and A. Weingessel. The quadprog package, version 1.5-8, 2019. Available electronically at https://CRAN.R-project.org/package=quadprog.
